# Building resilience through daily smartphone app use: results of a pilot study of the JoyPop app with social work students

**DOI:** 10.3389/fdgth.2023.1265120

**Published:** 2023-11-20

**Authors:** Katherine Maurer, Mert Kimyaci, Katy Konyk, Christine Wekerle

**Affiliations:** ^1^ School of Social Work, Centre for Research on Children and Families, McGill University, Montreal, QC, Canada; ^2^ Department of Pediatrics, McMaster University, Hamilton, ON, Canada

**Keywords:** resilience, affect regulation, social work, mHealth app, adverse childhood experiences, traumatogenic experiences, stress response

## Abstract

**Background:**

The JoyPop™ smartphone app is a digital intervention designed to enhance day-to-day resilience in youth, particularly those exposed to traumatogenic events [adverse childhood experiences (ACEs)]. Processes of adaptation that foster resilience in response to high stress include affect, cognitive, and behavioral regulation, and social interaction. Digital interventions have application for youth and those who provide them support, including social work trainees navigating the stressors of university studies concurrent with practice internships. Research on resilience-enhancing apps is needed to understand the underlying mechanisms by which change occurs and who is most likely to benefit from these interventions.

**Methods:**

Social work student participants (*N* = 91) were invited to use the JoyPop app two times daily for 28 days. Baseline ACE exposure and change-over-time in affect regulation, stress responsivity, and social support were evaluated after 2 and 4 weeks of app use with *t*-tests and generalized estimating equation (GEE) modeling.

**Results:**

Participants identified predominantly as cisgender women of European descent, mean age 26 years (SD = 6.78), 70% undergraduates, and reported consistent daily app use (*Mean days* = 26.9, SD = 1.90). Self-reported baseline ACE exposure was high (30% ≥ 5+). We tested change-over-time with generalized estimating equation and saw improvement in affect regulation in the Abbreviated Dysregulation Inventory scale (*β* = −3.38, *p* *=* <.001), and subscales of behavioral (*β* = −1.63, *p* *=* <.001), affect (*β* = −3.24, *p* *=* <.001), and cognitive regulation (*β* = 1.50, *p* *=* .009). Perceived stress decreased with app use (*β* = −2.65, *p* *=* <.001) and even more so for participants with reported exposure to more than 4 ACEs (*β* = −3.786, *p* *=* .030).

**Conclusions:**

The exploratory findings from our pilot study suggest that consistent use of the app may enhance multidimensional resilience amongst university students who self-report higher than average levels of baseline traumatogenic exposures. Our findings support an approach modeling resilience as a complex, dynamic, multicomponent process supported by resources within and between individuals. Further testing of the mechanisms of adaptation in response to high stress that enhance resilience and identification of the JoyPop™ app features that influence this change is needed to validate that daily app use could help youth with experiences of past and current high stress to better regulate their affect, reduce stress reactivity, and increase resilience.

## 1. Introduction

Exposure to elevated or exceptional stressors has the potential to cause psychophysiological change, i.e., is traumatogenic, and can inhibit well-being-promoting adaptations to stress across the lifespan, i.e., resilience. Resilience is comprised of complex interdependent contextually adaptive responses to high stress and adversity, which optimally enhance well-being (1). Although there are multiple definitions of resilience [see (2–4)], salient stress response processes include affect, cognitive, and behavioral regulation, as well as social interaction (1, 4). Interventions designed to promote individual resilience are anticipated to be more effective if they target multiple resilience processes.

Smartphone apps that claim to improve psychophysiological well-being or enhance resilience are increasingly available. Yet, many apps are not substantiated by research exploring their efficacy and effectiveness (5–8). Existing research predominantly tests mental health-specific apps (8–11). The JoyPop™ app is a digital intervention designed to help youth, particularly youth exposed to traumatogenic events [e.g., adverse childhood events (ACEs)], to enhance day-to-day resilience (12–14). This smartphone app combines digital accessibility and variety, offering resilience-enhancing activities across multiple evidence-informed resilience domains including self-regulation, attention enhancement, self-reflection, and social engagement. An equally complex and evidence-informed approach is needed to evaluate the effectiveness and efficacy of the multiple psychophysiological interventions included in the JoyPop™ app in the context of enhancing resilience for vulnerable youth.

### 1.1. Traumatogenic events and the potential psychophysiological effects of exposure

Across the lifespan, exposure to exceptional stressors and chronic adversity has the potential to effectuate psychophysiolological change (i.e., trauma) and influence the development of health-sustaining behaviors (4, 15–17). Exposure to exceptional stress is a precondition of resilience (1–3). However, exposure to traumatogenic stressors does not guarantee that individuals will experience sustained psychophysiological alterations (1, 2, 17). Thus, in resilience-focused research, it is imperative to assess exposure to traumatogenic events and experiences, as well as test for evidence of stress response adaptations that may be contextually and culturally well-being inhibiting. In the decades since the report of findings from the initial ACE survey (18), the link between retrospective report of childhood exposure to traumatogenic childhood experiences and physical and mental health outcomes in adulthood has been extensively explored (19–23). The ACE Questionnaire [ACEQ, (18)] identifies exposure to a list of 10 traumatogenic stressors during childhood including physical, emotional, and sexual abuse, neglect, caregiver substance abuse, mental illness, and intimate partner violence. Other measures, for example the Life Events Checklist for *DSM-5* [LEC-5, (24)], include a broader scope of traumatogenic stressors and events relevant to current well-being, e.g., environmental or man-made disasters, serious accidents, bodily harm, and adult experiences such as combat-related events. Quasi-experimental research in the social sciences has a limited ability to test the full range of potential causal factors related to psychophysiological phenomena such as trauma or resilience. Given this limitation, it is important to measure traumatogenic exposure complexly, beyond the ACEQ (18) binary structure (exposure: yes/no), and to include assessment of frequency, chronicity, severity [e.g., CTQ, (25)], and proximity [e.g., LEC-5, (24)] to account for a density of exposure factors when conducting causal intervention research (19–21, 23, 26).

In addition to measuring traumatogenic exposure complexly, testing for the presence of effects of exposure is essential. The ACEQ (18), and other traumatogenic risk-assessing measures, has been much criticized for conflating exposure with effect [e.g., (27–31)]. Exposure to any of the traumatogenic events measured in the ACEQ and LEC-5 can contribute to psychophysiological adaptations that result in, for example, posttraumatic stress disorder (PTSD), major depressive disorder (MDD), substance abuse, and physical health issues (21, 23, 32–38). Yet, in general population samples, only a small-to-moderate proportion of people reporting lifetime traumatogenic exposure also report adulthood clinical levels of major mental health diagnoses. For example, in a Canadian sample (*N* = 2991), 9.2% of respondents with multiple ACEs reported lifetime clinical levels of PTSD. Furthermore, 74% reported comorbid depression in population-level research (39).

Historically, the effects of exposure to exceptional adversity, e.g., child abuse, have been considered as injury [i.e., trauma, (33, 34, 40)]. Research has long focused on outcomes, frequently behaviors, which are pathologized and stigmatized as non-normative. Rapid expansion of the neurosciences led to a shift from psychological and sociological explanations and outcomes to brain-based process and mechanism-focused explanations of changes in well-being associated with adversity exposure. Neuroscience research has identified and validated many underlying mechanisms by which traumatogenic event exposures elicit change to brain structure and function, as well as behavior, proximally and distally [e.g., (15, 41–43)]. This research demonstrates that stress reactivity is a process of adapting to specific environments via change in brain structure and function (plasticity and epigenetics) rather than a process of being injured or damaged by exposure to high stress (44, 45). The psychophysiological capacity to adapt to the current environment is essential for human survival. Thus, the relationship between adaptation and well-being is entirely context-dependent. Adaptations themselves are value-neutral. It is only in relation to a specific environment that the capabilities of an adaptation to sustain or inhibit well-being (i.e., resilience) can be assessed (15, 16, 44–46).

For example, reduced hippocampal volume is a psychophysiological change to the stress-response system common to individuals living with PTSD (40, 47, 48) that can contribute to misinterpreting how dangerous or safe a situation may be, which in turn can foster paranoia, hypervigilance, and an overly reactive stress response (40, 48). If a person continues to live in an environment of exceptional stress, for example a youth in a situation of chronic abuse, hypervigilance and over-estimating level of threat can be very protective and help them to evade future harm. In contrast, in a low-stress environment, these adaptations can be highly disruptive to social engagement and functioning; for example, in a classroom, where cognitive attention is needed for learning, executive function in the brain may be hijacked for assessing the environment for safety precisely because the body's stress-response system adaptations are guided by the past, not the present (45). Hence, an adaptation that is resilient in the home can inhibit resilience and well-being in other environments [e.g., (49, 50)].

The neurophysiological mechanisms and processes of adaptation to stress are complex and dynamic. Interrelated psychophysiological processes, including cognition (e.g., attention) and affect (physiology and emotion) regulation, driven by epigenetics and repetition, effectuate changes to neuronal pathways, brain structure, and stress response set points (brain plasticity). In turn, these changes (adaptations) influence proximal and distal biological, psychological, behavioral, and social stress responses in a continual looping feedback of a person's interaction with their internal and external environment (15, 16, 33, 40, 41, 50). Cognitive, affective, and behavioral adaptations to ensure future safety are formed to meet the threats of the past and become entrenched (habitual) stress response patterns in the present through repetition (1, 42–45, 50). Thus, exposure to a particular traumatogenic event does not have to be chronic for adaptations to occur if the stress response to the event continues to be repeated in the face of present stressors (40, 50).

For example, a person who experiences a car accident will naturally experience vulnerability when driving again. If that vulnerability elicits a strong stress response and overestimation of threat repeatedly, the person may develop an inability to drive in the future due to an elevated anticipatory threat response. This self-reproducing cycle of past experience-oriented adaptation to prepare to respond to future stress underlies the relationship between ACEs and adult mental and physical well-being. Furthermore, the entrenchment through repetition process is the basis of intergenerational trauma within families, communities, and cultures that experience past and current high adversity in the form of interpersonal and structural oppression, abuse, and violence (1, 50–54). Vulnerability and resilience are not solely individual processes. Affect regulation is the principal mechanism of stress adaptation and is thus central to overall well-being (15, 44, 52, 55). Difficulties with affect regulation, as may result from exposure to multiple or exceptional stressors, habituated through repetition, can inhibit the development of health-sustaining adaptations, particularly during adolescence (56–59), and affect resilience over the lifespan (15, 44, 52, 55, 60).

### 1.2. Affect regulation: a process model

Affect regulation is a complex, multidimensional process that involves the modulation of “internal physiological, emotional, cognitive, and behavioural responses to external and internal stress (affect arousal)” (49). Exposure to high stress and adversity, particularly to multiple adverse events or those that are frequent and/or severe, have substantive potential to generate persistent states of affect dysregulation (44, 55). When an individual experiences dysregulation, cognitive processes that support the maintenance of or return to equilibrium are limited or fully inaccessible (15, 55, 61). Lack of regulation capacity can lead to chronic affect hypo-arousal, manifesting in social disengagement, depression, depersonalization, and at the extreme, dissociation. Conversely, hyper-arousal dysregulation is associated with impulsivity, aggression, and violence (43, 61). Thus, stress response adaptations can play a debilitating role in limiting biological, psychological, and social well-being and resilience in childhood, youth, and throughout adulthood (15, 16, 50, 51, 62). Intervention following traumatogenic exposure has the potential to prevent stress adaptation entrenchment or may engender adaptations that enhance and strengthen affect regulation capacity, and consequently resilience, through repetition (15, 22, 46, 50, 55, 56, 59, 63).

**Figure 1:**
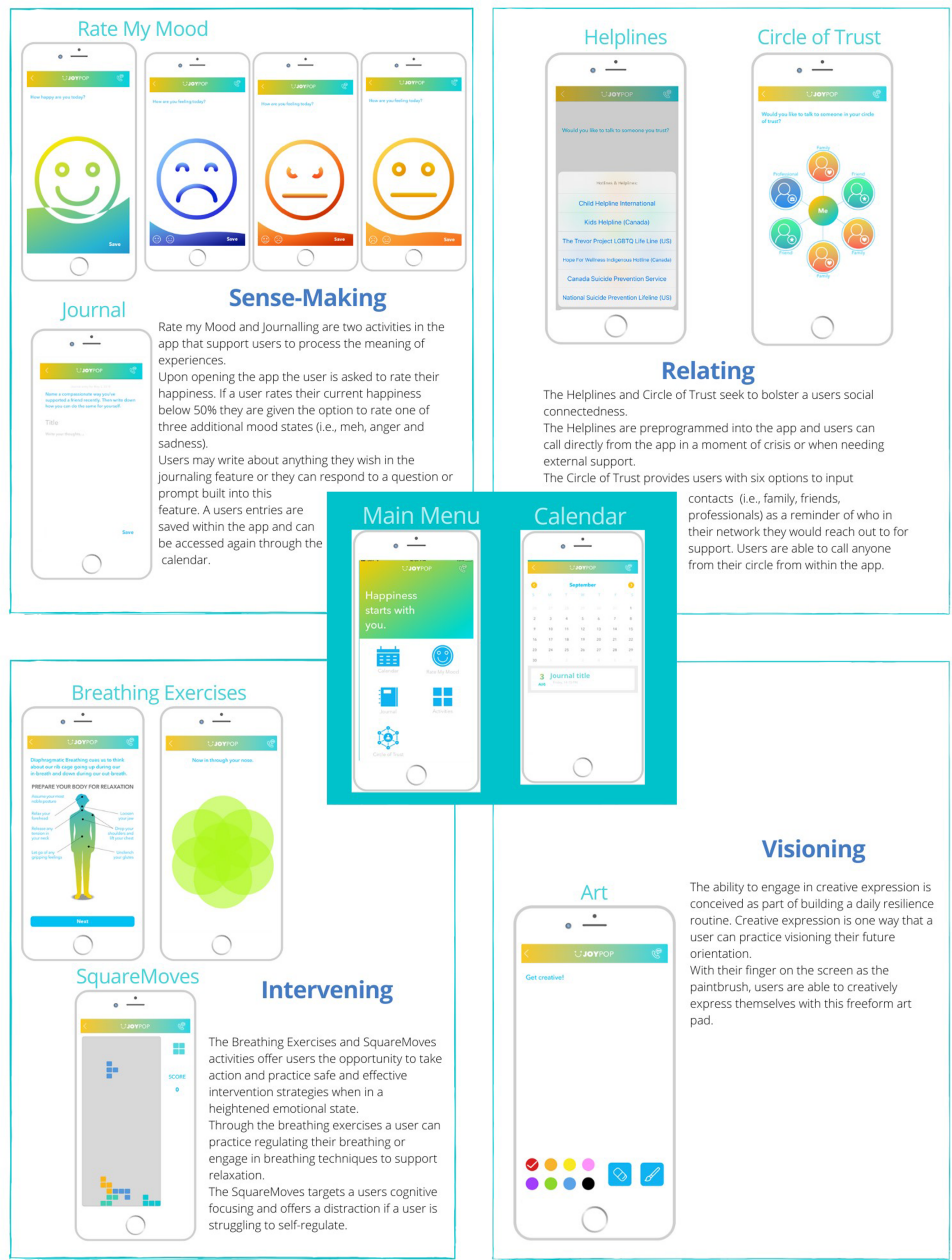
Activities and the resilience domains in the JoyPop™ app.

Given the primacy of affect regulation in both metal health and resilience, a large body of multi- and interdisciplinary theoretical and empirical literature supporting focuses on affect, which combines physiological, emotional, and cognitive regulation processes, and modeling regulation as a multi-phase process (15, 44, 46, 56). Thus, Gross et al. (55) propose a process model of four strategies and four stages of affect regulation to inform research and interventions. The four stages are: (1) Identification: Ascertaining if there is a need to change current affect or not; (2) Selection: choosing a regulation strategy to change affect as identified in the first stage; (3) Implementation: decisions about taking actions inherent to the regulation strategy identified in stage two to change current affect; (4) Monitoring: ongoing iterative updating of the three previous stages to decide to continue on with the affect change via a chosen regulation strategy and its implementation, change to another strategy, or stop regulation efforts (55). The design and structure of the JoyPop™ app mirrors the staged regulation process outlined by Gross et al. (55) starting with mood rating followed by the option to choose amongst multiple features to engage in a variety of regulation activities [Figure 1 (64),] with the opportunity to change activity or stop using the app at any time.

As outlined below (see section 1.4), the activities available in the JoyPop™ app support the development of the four different affect regulation strategies in Gross et al.'s (55) model: situational, attentional, cognitive, and response modulation. Situational strategies include modifications to the external environment, whereas attentional strategies are those that divert focus from one aspect of a situation to another. Cognitive strategies seek to regulate affect through efforts to change thoughts or perceptions of a situation, and response modulation strategies target affect through behavioral or physiological changes (p. 132). Not only are multiple strategies needed to regulate affect but regulatory flexibility is also necessary to be able to employ the strategies adaptively, given a specific or shifting environmental context (55, 56, 65–68). The concept of envisioning changes in affect regulation capacity following high adversity exposure as environmentally adaptive and as a process have emerged from neuroscience and stress physiology identification of the mechanisms of adaptation (15–17). This interdisciplinary research demonstrating the centrality of adaptation to environment has facilitated a conceptual shift from regarding affect regulation capacity as a trait to acknowledging it as a complex and dynamic state (43, 44). Furthermore, the emphasis on flexibility in adaptive capacity rather than pathologizing regulation patterns as an individual trait or characterologically-driven strategy (i.e., antisocial behavior) centers the evaluation of a stress response adaptation on sustaining well-being as the marker of resilience (1, 46, 52). The assessment of stress response processes, including affect regulation capacity and other adaptive responses, as adaptive to specific environments which may not be well-fitted to other environments is essential to the definition and assessment of resilience and resilience-enhancing interventions such as the JoyPop™ app.

### 1.3. Resilience and affect regulation

The definition of resilience as a complex dynamic biopsychosocial adaptive process centers the dependence on access to resources throughout the social environment in ways that are culturally meaningful to individuals, families, and communities to enhance well-being and foster resilience (1, 15, 16, 52, 68). Barriers to access to resources can be structural and systemic as well as internal to individuals in the form of challenges to maintaining psychophysiological well-being such as limited capacity to regulate affect in the face of stressors (15, 16, 55). Thus, affect regulation is a core resilience process. As an intervention, the JoyPop™ app provides access to resources that can be engaged to enhance affect regulation capacity, cognitive function, physiological, and behavioral regulation that may serve to bolster individual resilience to high stress and adversity, a precondition of resilience. In turn, the capacity to regulate affect flexibly when experiencing adversity may support individuals, groups, and communities to navigate to resources to change the conditions of adversity and negotiate for those resources “to be provided and experienced in culturally meaningful ways (Ungar, 2011, p. 10)” (44–46, 51, 52). The JoyPop™ includes evidence-informed features to foster increased affect regulation capacity and flexibility to enhance resilience (12–14).

### 1.4. The JoyPop™ app: a resilience enhancing intervention

Resilience, as a multicomponent concept, can be enhanced at any time. In addition to changes in environmental stressors, capacities such as affect regulation skills are responsive to interventions at any life stage. However, the development of regulation capacity is a primary developmental task during adolescence and emergent adulthood. Thus, including affect regulation in resilience interventions for youth may be particularly effective (56–59). The JoyPop™ app is designed to enhance resilience by providing an array of activities (interventions) that help users manage elevated levels of stress in the moment and adaptively over time through repeated use of app activities (12–14).

As noted, the app was designed to target four domains of resilience: sense-making, relating, visioning, and intervening [see Figure 1, (64)]. Although not designed with it in mind, the JoyPop™ app is commensurate with Gross et al.'s (55) process model of four strategies and four stages of affect regulation. Upon opening the app, user are prompted to rate their mood (Identification Stage). Subsequently, users can select (Selection Stage) from an array of activities that through use can enhance affect regulation capacity via repetition (entrenchment) of specific affect regulation strategies (Implementation Stage). For example, the Circle of Trust supports users to reach out to someone in their social network (situational strategy), SquareMoves offers a distraction from overwhelming cognitions (attentional strategy), journaling provides an opportunity to reevaluate a situation (cognitive strategy), and the breathing exercises support physiological changes in arousal [response modulation strategy, (55)].

The opportunity to independently select an activity in the JoyPop™ app supports the process approach that Gross et al. (55) outlined to facilitate active engagement in choosing and practicing regulation strategies that are adaptable to the app user's current needs. For example, if a person would commonly reach out for social support when experiencing activation of their stress response system yet are in a situation in which they cannot immediately speak to someone, they have the option to access another affect regulation strategy in that moment, such as diaphragmatic breathing or SquareMoves. The ability for users to select a regulation activity based on differing needs and contexts may foster the development of the important capacity of affect regulation flexibility over time (65–68). Repeated use of app features to engage in the four stages and multiple strategies to regulate affect (55) can lead to the entrenchment or adaptation of stress responses that enhance well-being and engender flexibility grounded in affect regulation strategies that modulate or inhibit elevated stress reactivity. Qualitative reports of user experiences with the JoyPop™ app suggest that users appreciated the flexibility embedded in the design of the app and perceived changes in their capacity to regulate their affect (14, 64). The app has also undergone a process of user-led revisions of the features to increase cultural relevance for indigenous youth (69, 70).

### 1.5. Piloting the the JoyPop™ with social work trainees

The experience of training to become a social worker can result in psychological distress (71). Social workers commonly train and will eventually be employed in environments with high levels of stressor exposure (i.e., child welfare, health care). Trainees and experienced social workers alike are at risk for developing burnout, compassion fatigue, and traumatic stress reactions (71–77). Social workers are not just vulnerable to stressors in the workplace, as many have been exposed to previous traumatogenic stressors, which may serve as motivation to enter the profession (78, 79). However, previous traumatogenic exposures may increase risk for psychological distress and secondary traumatic stress reactions (80) and even impact their ability to practice effectively (78, 81). Thus, social workers can benefit from developing a toolbox of stress management skills to augment their individual resilience and capacity to remain engaged in their work.

During their university training, social work students are taught to engage in critical self-reflection to identify and confront assumptions in their identity and cultural values that impact their practice (82). Engaging in self-reflective practice supports social workers to develop self-awareness and regulation skills that enhance their capacity to engage in culturally safe ethical practice (83), maintain empathy, prevent the development of psychological distress, burnout, and secondary traumatic stress (84), and foster resilience (71).

The primary aim of the pilot study of the JoyPop™ app with social work trainees was to evaluate whether or not using the app consistently over time would enhance resilience with a sample of predominantly young people exposed to current and past stressors. Piloting the new intervention with social work students allows us to test the app under conditions of elevated stress exposure (university life) with a population that has access to many social and psychological supports. The current analysis explores three components of resilience: affect regulation, stress response, and perceived social support. Using repeated measures testing, we specifically examined (1) if JoyPop™ app use is associated with reduced affect dysregulation, reduced stress response, and increased perceived social support; and (2) whether changes in affect regulation capacity, stress response, and perceived social support of social work students across the three measurement time points are associated with pre-study traumatogenic events (ACEs) exposure.

## 2. Materials and methods

### 2.1. Design

**Figure 2:**
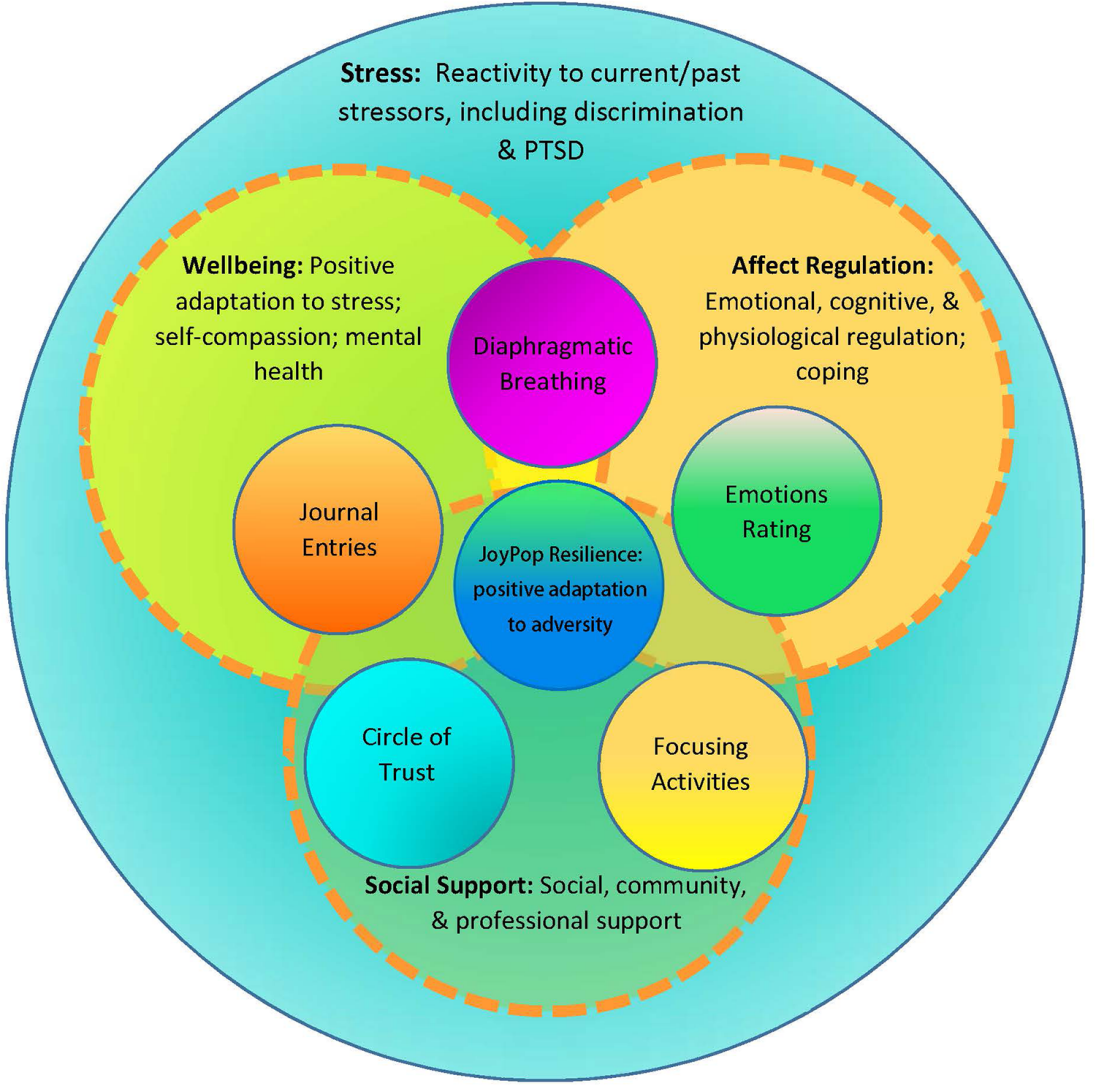
Conceptual model of four domains resilience: affect regulation capacity, stress response, wellbeing, and social support.

This quantitative analysis is part of a mixed-methods pilot panel study evaluating the JoyPop™ app. Data were collected on demographics, prior traumatogenic exposure, and a multi-factor conceptual model of resilience that included four domains relevant to the app features: affect regulation capacity, stress response, well-being, and social support (see Figure 2). Study participants completed online surveys at three time points: before app use, at 14 days of app use, and after 28 days of app use (see 14 for the study protocol). Ethics approval was received from the authors' respective universities. Qualitative interviews exploring user experience of the JoyPop™ app were conducted following the 28-day pilot use period [see (64)].

### 2.2. Data collection and participants

The participants were recruited from the social work department of a Canadian university between September 2019 and October 2020. Participant eligibility criteria were enrollment in social work department, English proficiency, and daily access to an iOS mobile device with internet connectivity. Prior to the COVID-19 pandemic health protocols, participants were recruited via posters and flyers, as well as direct recruitment in social work course sessions. After pandemic safety measures were implemented, we shifted to online recruitment using social media platforms and virtual classrooms. Consent to participate in the study was obtained in person pre-pandemic and via a signed PDF during the pandemic. To mitigate any risks that might be associated with reporting on traumatogenic or distressful event exposure, a trained research assistant was present, in person and virtually, while participants responded to surveys. Additionally, participants were provided with contact information for accessible mental health support services.

Participants completed all surveys on Lime Survey, a web browser-based platform, at three time points during the 28-day use period. Detailed instructions on using the JoyPop™ app prior to starting the pilot period were given to all participants, who were instructed to use the app at least twice a day, preferably in the morning and evening, for 28 days, starting the day after they completed the baseline survey. Email reminders were sent twice daily. Participants used a 4-digit randomly assigned number to log into the JoyPop™ app. App use data were stored in an encrypted web hosting service and accessed by the research assistants via MySQL, an encrypted and password-protected database storage center. Anonymized app use and survey data were housed on an encrypted cloud-based hard drive.

Participants were compensated $20 for each of the first two surveys and $30 for the final survey. Starting in 2020, we offered the option of receiving course credit instead of monetary compensation to students in several required undergraduate and graduate social work courses. Of the 45 participants who completed the study pre-pandemic, 11 (24.4%) received course credit. After March 2020, 55 of 58 participants (95%) received course credit.

### 2.3. Measures

Including the ACEQ (18), three measures of traumatogenic exposure were administered in the first wave of data collection. We created a variable to measure daily app use (DAU). An additional 24 measures were administered across all three of time points. We conceptualized these into four subcomponents of resilience: affect regulation capacity, stress, well-being, and social support (see Supplementary Materials). The conceptual model included covariates taken from sociodemographic characteristics of gender identity, ethnicity, socioeconomic status, and baseline substance use. To explore our research questions, we included scales that measure affect regulation capacity, stress response, and social support (three panels) and lifetime exposure to traumatogenic events measured at baseline.

#### 2.3.1. Daily App Use (DAU)

We created a binary independent variable (IV) of the aggregate count of DAU over the 28-day study period to test the effect of repeated use of the app. Each time a participant signed into the app and rated their mood or engaged in an activity, the date and use timespan were logged. We aggregated these data to create a daily score of 1 if participants rated their mood or engaged with any feature for 30 s or more per 24-hour period.

#### 2.3.2. Baseline measures

##### 2.3.2.1. Adverse Childhood Experiences Questionnaire (ACEQ)

The ACEQ (18) is a dichotomous 10-item self-report measure assessing abuse, neglect, and household dysfunction experiences during childhood included only in the baseline survey. We added a response option to capture chronicity of exposure: “never” (coded as 0), “at least once” (coded as 1) and “many times” (coded as 2). We added an item to capture maternal violence towards the father, given the prevalence of bidirectional partner violence in community samples (85). Responses were summed for a score range of 0 to 22. The original ACEQ was shown to have a good construct validity compared to other traumatogenic events exposure measures and good test-retest reliability (86, 87). Internal consistency in our sample was good (*α* = .78).

##### 2.3.2.2. Childhood Trauma Questionnaire-Short Form (CTQ-SF)

The CTQ-SF (25) is a 28-item self-report measure of maltreatment history in childhood and adolescence included only in the baseline survey. It is composed of five subscales based on the type of maltreatment—physical, sexual, and emotional abuse, and physical and emotional neglect. Each subscale contains five items, plus an additional three items on minimization and denial to assess underreporting. The Likert scale has five response options—“never true = 1”, “rarely true = 2”, “sometimes true = 3”, “often true = 4”, and “very often true = 5”—to measure participants' childhood experiences of abuse and neglect. Subscale and total scale scores were summed for a total score that ranged from 25 to 125. The short version of the CTQ demonstrated good criterion-related validity (25). The internal consistency in our sample for total CTQ-SF score was *α* = .919 and ranged between *α* = .546–.941 for the subscales.

##### 2.3.2.3. Life Events Checklist for DSM-5 (LEC-5)

The LEC-5 (88) is a 17-item self-report measure of lifetime traumatogenic events exposure. The LEC-5 item events include sexual and physical assaults, environmental or man-made disasters, serious accidents, bodily harm, and combat-related events. Individuals were asked to indicate whether they experienced the event personally, witnessed the event, or learned about it happening to someone else and if the exposure was part of their job. Responses to each item were summed to calculate a subscale score (0–17) for each exposure category. A total LEC-5 score was calculated by summing the scores of “experienced it,” “witnessed it,” and “was part of job” subscales. The LEC-5 has reasonable test-retest reliability and good construct validity (89). The internal consistency of “experienced it” and “witnessed it” was reasonable (*α* = .652 and.697). The internal consistency of “learned about it” and “was part of job” was good (*α* = .839 and .848).

#### 2.3.3. Panel measures: affect regulation capacity measures

##### 2.3.3.1. Abbreviated Dysregulation Inventory (ADI)

The ADI (90, 91) is the shortened version of a measure of psychophysiological dysregulation originally designed to assess risk of substance use disorder. This 30-item self-report multidimensional scale consists of three subscales: affective, behavioral and cognitive dysregulation. The response options, scored 0–3 on a Likert scale for the timeframe of the previous 2 weeks, are “never true”, “occasionally true”, “mostly true”, and “always true”. The aggregate ADI score (0–90) and each of the subscale scores (0–30) were calculated by summation. The full ADI and subscales demonstrated construct validity in relation to aggression and quality of life in an adolescent sample. The ADI also possesses good test-retest reliability (90). Internal consistency of the total ADI scale was *α* = .863 and the subscales ranged from *α* = .810 to.839. Internal consistency of the ADI measure at mid-study was *α* = .836 and *α* = .900 at post-study.

##### 2.3.3.2. Difficulties in Emotion Regulation Scale-Short Form (DERS-SF)

The DERS-SF (92) is an 18-item self-report measure of emotion regulation problems which includes six subscales: awareness, clarity, nonacceptance, goals, impulse, and strategies. The response options for the DERS-SF are on a scale of 0%–100%: “almost never (0%–10%)”, “sometimes (11%–35%)”, “about half the time (36%–65%)”, “most of the time (66%–90%)”, and “almost always (91%–100%)”. The responses were coded as 1, 2, 3, and 4 respectively for scoring purposes. The participants were asked to respond referring to the past 2 weeks. Scores were summed for scale and subscale item responses. The DERS-SF has good test-retest reliability and adequate construct validity, comparable to the long version measure (93). The DERS-SF (*α* = .863) scale and all seven subscales (*α* = .826–.948) demonstrated good internal consistency at baseline. The internal consistency of the DERS-SF at mid-study was *α* = .908, and *α* = .911 at post-study.

##### 2.3.3.3. Executive Functioning Index (EFI)

EFI (94) is a 27-item measure of self-rated executive function, comprised of five subscales—motivational drive, organization, strategic planning, impulse control, and empathy. Likert scale response options ranged from “1 = not at all” and “5 = very much”, the midpoint 3 was described as “somewhat”. Scores were summed for scale and subscale item responses. The EFI has demonstrated good construct validity through correlations in relation to several self-rating executive-function measures (94). The EFI showed acceptable internal consistency at pre-study (*α* = .703), mid-study (*α* = .771) and post-study (*α* = .791) testing.

##### 2.3.3.4. Positive and Negative Affect Schedule (PANAS)

The PANAS (95) is a collection of two self-report mood scales each composed of 10 items descriptive of positive or negative affect. Participants used a Likert scale to rate affect items based on their experiences of the past 2 weeks coded as “very slightly or not at all = 1”, “a little = 2”, “moderately = 3”, “quite a bit = 4”, and “extremely = 5”. A total score was created for Positive Affect and Negative Affect by summing 10 items belonging to that mood. The PANAS was shown to have excellent correlation with longer measures of underlying mood factors (95). Both Positive Affect and Negative Affect showed good internal consistency at baseline, *α* = .887 and *α* = .884 respectively. The internal consistency of the PANAS Positive Affect was *α* = .896 at mid-study and *α* = .881 at post- study. The internal consistency of the PANAS Negative Affect was *α* = .891 at mid-study and *α* = .893 at post-study.

##### 2.3.3.5. Patient Health Questionnaire-9 (PHQ-9)

The PHQ-9 (96) is a 9-item self-report diagnostic measure of depression severity. Each item corresponds to the diagnostic criteria for major depressive disorder in the DSM-IV. Participants rated how often they have been bothered by an item in the past 2 weeks using a three-point Likert scale coded as “0 = not at all”, “1 = several days”, “2 = more than half the days”, and “3 = nearly every day.” A total depression severity score was calculated by summing all the items. The PHQ-9's construct validity was shown with quality of life, health care utilization, and symptom-related difficulties (97). The internal consistency of the PHQ-9 measure in our study in all three waves was strong (*α* = .847,.834 and.838).

#### 2.3.4. Panel measures: stress responsivity measures

##### 2.3.4.1. PTSD Checklist for DSM-5 (PCL-5)

The PCL-5 (98) is a 20-item self-report measure of PTSD symptoms as they are conceptualized in the DSM-5. Each item is designed to represent a symptom of PTSD, which are organized into four clusters — cluster B (items 1–5), cluster C (items 6–7), cluster D (items 8–14), and cluster E (items 15–20). We added two items to the PCL-5 that assess depersonalization/derealization symptoms (99). Participants indicated how much they have been bothered by a symptom in the past 2 weeks by choosing from a 4-point Likert scale with the options “not at all”, “a little bit”, “moderately”, “quite a bit” and “extremely”. These response options were coded from 0 to 4, respectively. We calculated a total score by summing the item responses. We calculated summed scores for the clusters, the original measure, and the 22-item amended checklist. The PCL-5 was shown to have strong convergent validity with another PTSD symptom severity scale and its subscales (100) and to have good test-retest reliability (98). The internal consistency of both the 20-item (*α* = .929) and the 22-item (*α* = .933) measures were strong at baseline, as was internal consistency of the 22-item scale at *α* = .943 mid-study and post-study.

##### 2.3.4.2. Perceived Stress Reactivity Scale (PSRS)

The PSRS (101) is a 23-item self-report measure of an individual's perceived typical response to everyday stressful situations. The scale is composed of five subscales — reactivity to work overload, prolonged reactivity, reactivity to social conflict, reactivity to social evaluation, reactivity to failure. Participants reported their reactions to specific situations over the past 2 weeks. There are three response options for each item, worded in accordance with each item and coded as 1, 2 and 3 with 1 representing the least reactivity and 3 the most reactivity. Several items were reverse-coded. The total PSRS score and subscale scores were summed. The PSRS was shown to have construct validity with self-efficacy, neuroticism, chronic stress, and perceived stress (101). The internal consistency of the PSRS measure for our sample at all three time points was strong at pre-study (*α* = .815), mid-study (*α* = .870), and post-study (*α* = .874).

##### 2.3.4.3. Brief Coping Orientation to Problems Experienced Inventory (Brief COPE)

The Brief COPE (102) is 28-item self-report measure of coping responses to stressful events. The Brief COPE is composed of three subscales each of which is an overarching coping style — emotion-focused, problem-focused, and avoidant. The participants were instructed to rate how much or how frequently they were using each of the coping strategies over the past 2 weeks. The response options were, “I haven't been doing this at all = 1”, “I’ve been doing this a little bit = 2”, “I’ve been doing this a medium amount = 3”, and “I’ve been doing this a lot = 4”. A score was calculated for each of the subscales by summing the items belonging to that coping style. The long version of the Brief COPE scale was shown to have acceptable test-retest reliability (103). An exploratory factor analysis demonstrated consistency between responses to the brief and long versions (102). The internal consistency at pre-study, mid-study and post-study for items of the problem-focused coping subscale was good (*α* = .859,.860,.871), but it was low for the emotion-focused coping subscale (*α* = .552,.686,.704) and avoidant coping subscale (*α* = .634,.663,.631).

##### 2.3.4.4. Connor Davidson Resilience Scale-10 (CD-RISC-10)

The CD-RISC-10 (104, 105) is the 10-item short version of the original 25-item self-report scale measuring resilience grounded in the biopsychosocial model. It was designed to be used with both community and clinical populations. We asked the participants to indicate how much they agree with each of the 10 items as it applied to them over the last 2 weeks. If a particular situation had not occurred, the respondents were instructed to answer according to how they think they would have felt. The response options and the associated scoring codes were “not true at all = 0”, “rarely true = 1”, “sometimes true = 2”, “often true = 3”, and “true nearly all the time = 4”. We calculated a total CD-RISC-10 score by summing response codes of all items, which ranged from 0 to 40. Across different populations, the long version was shown to have good test–retest reliability and construct validity relative to a perceived stress measure (*r* = 0.76) (104). Scores on this 10-item version were highly correlated with the scores on the long version (*r* = .92) (105). The CD-RISC-10 in our pre-study, mid-study and post-study surveys demonstrated good internal consistency (*α* = .859,.840,.863).

#### 2.3.5. Perceived social support measures

##### 2.3.5.1. Multidimensional Scale of Perceived Social Support (MSPSS)

The MSPSS (106) is a 12-item self-report measure of perceived social support adequacy from family, friends, and significant others. The participants were instructed to respond to this scale in all three surveys by indicating how they felt about each of the 12 items for their experiences over the past two weeks. The response options and their relevant scoring codes were: “very strongly disagree = 1”, “strongly disagree = 2”, “mildly disagree = 3”, “neutral = 4”, “mildly agree = 5”, “strongly agree = 6”, and “very strongly agree = 7”. The MSPSS score was calculated by finding the mean of the sum score of the 12-items. MSPSS was shown to have good test-retest reliability and moderate construct validity (106). The internal consistency of the MSPSS measure for our sample at all three time points was strong (*α* = .899,.925,.911).

##### 2.3.5.2. Perceived Community Support Questionnaire (PCSQ)

PCSQ (107) is a 14-item self-report measure that assesses perceived community support through three dimensions—community integration, community participation and use of community organizations. In all three surveys, participants were asked to rate how they felt about each statement in the items for their experiences over the past 2 weeks by choosing one of “strongly disagree = 1”, “disagree = 2”, “neither disagree nor agree = 3”, “agree = 4”, and “strongly agree = 5”. A total perceived community support score was calculated by summing all the responses (107). The PCSQ was shown to have factorial validity (108). The internal consistency of the PCSQ measure was good at all three time points of pre-study (*α* = .899), mid-study (*α* = .914) and post-study (*α* = .931).

### 2.4. Analytic plan

We conducted our data analysis using SPSS 29 (IBM Corporation). Given that data were collected under two circumstances that might influence resilience—in person pre-COVID and virtually during the pandemic social distancing measures—we first explored, using two-sample *t*-tests, if there were any significant differences on key variables for the analysis of affect regulation, stress, and social support, in addition to basic sociodemographic characteristics. The during-COVID sample included more first-year Bachelor's in social work (BSW) students (84% v. 54%) and a statistically significant difference on PHQ-9 scores of less than 1 standard deviation between the two groups. We found no significant differences in the 12 participants excluded from this analysis from those retained when compared on sociodemographic characteristics, pre-study traumatogenic exposure, or key affect regulation, stress response, or social support variables at baseline. Our missing data analysis showed that all variables contained 10% or less missing data. Thus, we were able to create a combined dataset (*N* = 91) with which we conducted Little's test (109) that confirmed that any missing data were missing completely at random (110).

We proposed a conceptual model of resilience comprised of constructs of affect regulation capacity, stress responsivity, well-being, and social support [see Supplementary Materials, (111)] as a process-focused rather than outcome-focused exploration of resilience in the evaluation of the JoyPop™ app features over the pilot study timeframe. Our IV of central interest, DAU, did not contain enough variance with a mean of 26.9 (*SD* = 1.90) for the 28-day observation period to test within-subject differences of the dependent variables (DVs). To maintain a focus on change-over-time, we chose generalized estimating equation (GEE) modeling (112) for the analysis. GEE is a marginal method for analysis of repeated measures data that produces regression estimates with both continuous and categorical variables having non-normal distributions. The marginal parameters GEE estimates are population-level averaged effects in contrast to the within-individual effects estimated in multi-level modeling. Thus, in GEE it is assumed that cases are dependent within subjects yet between subjects, at the population level, are independent. GEE is robust to autoregressive time-series repeated measures data such as we collected to model a population average longitudinally and generate parameter estimates, which are fixed effects (112, 113).

However, GEE does not handle even low levels of missing data well (113), so we imputed the dataset to account for person-mean missing items in questionnaires. Although imputation can inflate reliability estimates, given the low missing data (<10%) it is a tolerable risk (110). Imputation is a common method to accommodate monotone and intermittent missing data that is expected in longitudinal repeated measures testing (113). In addition, maximum likelihood was run as part of the GENLIN syntax within SPSS. Prior to running GENLIN for GEE, we restructured the imputed data set from long to wide/horizontal format to test dependent scale and subscale variables of interest at the three data collection time points with TIME as the index variable rather than as separate variables. Restructuring generates one variable per measure that includes the scores for each time point for each participant. Thus, a marginal score that represents the between-individuals difference rather than the within-individual difference over time of conditional modeling is generated (113). Using GEE modeling, we were able to test change-over-time of DVs as well as the interaction effect of TIME, DVs, and selected covariates.

**Figure 3:**
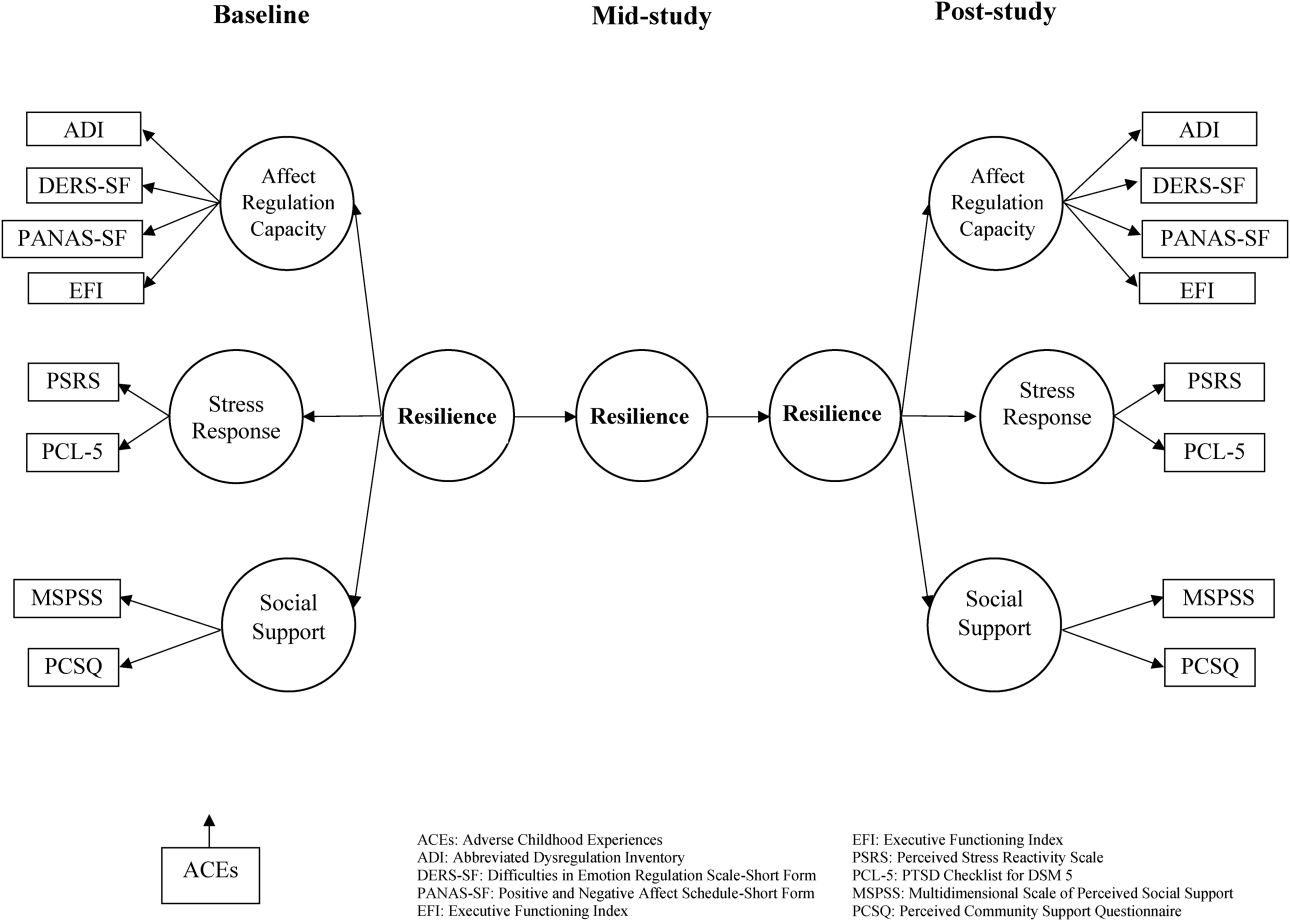
A smartphone intervention to promote brain health and resilience for social work students.

We conducted a preliminary analysis of bivariate correlations and *t*-tests to explore relationships between 15 measures relevant to three constructs the conceptual model (affect regulation, stress responsivity, social support) in the unimputed dataset prior to the main GEE analysis. Based on the bivariate analyses, we calculated *t*-tests amongst all the variables of interest that included ACEQ, CTQ, and LEC-5 as variables of traumatogenic exposure and the affect regulation variables (ADI, DERS-SF, EFI, PANAS, PHQ-9, Brief-COPE, CD-RISC-10), the stress responsivity variables (PCL-5, PSRS), and two variables of social support (MSPSS, PCS). Only a few significant relationships emerged, primarily on DV subscales, on our sociodemographic characteristics variables of age, gender, ethnicity, or level of education. Thus, we did not include any sociodemographic characteristics variables as covariates in our modeling. Based on the *t*-test results, we respecified our conceptual model to explore change-over-time on the domains of resilience of affect regulation, stress responsivity and social support with the indicator variable of TIME (replacing DAU) and pre-pilot scores on the three traumatogenic exposure variables as the IVs in a GEE analysis [see Figure 3, (111)].

## 3. Results

### 3.1. Participants

A total of 103 participants consented to be a part of the study. Of those 103, two did not complete any surveys, five dropped out before completing the post-survey measures, and another five were missing more than 10% of responses in one of the surveys. Thus, the final sample included 91 participants for a retention rate of 88.3%. The mean age of participants was 26 years old (SD = 6.78), with 57% of the sample 25 years old or less. Sample sociodemographic characteristics are presented in Table 1. Categories with two or fewer participants were combined into a category labeled “Additional” to protect confidentiality. Notably, of the 91 participants, 80% were cis women, 70% were undergraduate social work students, and 58% reported that their sexual orientation was heterosexual. A majority (76%) identified ethnically as European/European descent with the remainder identifying as Asian/South Asian/Southeast Asian, African/Black-Afro-Caribbean, Middle Eastern, Métis, and Latinx. Although 90% of the students were pursuing full-time studies, half reported that they were working part-time. The majority (70%) reported that they were not receiving services from a mental health professional during the study. The mean of app use days was 26.90 (SD = 1.90) out of 28 days of the pilot.

**Table 1:** Sociodemographic characteristics of participants at baseline.

Sociodemographic characteristic	Full sample	
	*n*	%
Ethnicity	89	
European/European descent	68	76.4
Asian/South Asian/Southeast Asian	8	9.0
Black-Afro-Caribbean	5	5.6
Métis	2	2.2
Additional ethnicity	6	6.7
Current year of social work study	91	
BSW	64	70.3
Graduate studies	27	29.7
Gender identification	90	
Cis woman	72	80.0
Cis man	10	11.1
Non-binary/ non-conforming	3	3.3
Trans man/ trans masculine, Genderqueer, not represented	5	5.6
Age (*m* = 26, SD = 6.78)	91	
19–25 years old	52	57.1
26–51 years old	39	42.9
Current educational status	91	
I am a full-time student	82	90.1
I am a part-time student	7	7.7
Additional	2	2.2
Household income	89	
$0–39 999	25	28.1
$40 000–99 999	32	35.9
$100 000 and up	32	35.9
Receiving mental health services	91	
No	64	70.3
Yes	25	27.5
Prefer not to say	2	2.2

Traumatogenic exposure descriptives at baseline are presented in Table 2. Of the 91 participants, 16 (17.6%) reported no ACEs, 48 (52.7%) reported having experienced 1 to 4, 27 (29.7%) reported 5 or more. A score of 4 or more occurs in 5%–10% of the general population, is considered clinically significant, and associated with inhibition of well-being long-term (20). The mean ACEQ score for the dichotomous version was 3.12 (SD = 2.52). Of the 75 participants who reported at least 1 ACE, 52 (57% of the sample) reported experiencing 1 or more ACEs multiple times. Both overall scale scores and several subscale scores (emotional abuse and neglect, sexual abuse) of the CTQ-SF were a standard deviation higher than population norms (114) on the five categories of abuse or neglect (25). As measured by the LEC-5, participants reported personally experiencing, witnessing, or experiencing as a part of their job, on average seven traumatic events in their lifetime. Although there is a dearth of normative data on the LEC-5 (115), mean self-reported exposure in our sample appears to be elevated [e.g., (116)]. Several measures of mental health were included in the surveys. At baseline, the mean score on the PCL-5 was 20.21 (*SD* = 14.80; Table 4), which is substantially lower than population means clinical levels for PTSD (scores of 28 to 37, 99). The mean PHQ-9 score at baseline for the sample was 7.23 (*SD* = 5.11) which is within the range (5–9) of population-level scores for mild depression (97).

**Table 2:** Traumatic exposure descriptives at baseline (*n* = 91).

Measure	Frequency	Mean (SD^a^)	Range
Adverse Childhood Experiences Questionnaire (ACEQ)			
ACEQ—yes/no response		3.12 (2.52)	0–9.0
Zero ACEs	16 (17.6%)		
1–4 ACEs	48 (52.7%)		
5 or more ACEs	27 (29.7%)		
ACEQ—frequency response		4.27 (3.89)	0–16.0
Never	16 (17.6%)		
At least once	23 (25.3%)		
Many times	52 (57.1%)		
Childhood Trauma Questionnaire (CTQ)			
CTQ total score		41.11 (14.39)	26.0–94.0
Emotional abuse		10.23 (4.99)	4.0–24.0
Physical abuse		6.31 (3.06)	4.0–23.0
Sexual abuse		6.81 (4.37)	5.0–25.0
Emotional neglect		9.71 (4.09)	5.0–20.0
Physical neglect		6.56 (2.21)	5.0–14.0
Life Events Checklist for DSM-5 (LEC-5)			
LEC total score		7.03 (2.21)	0–22.0
Happened to me		2.42 (2.05)	0–11.0
Witnessed		2.95 (2.51)	0–10.0
Learned about		5.93 (4.24)	0–16.0
At my job		1.67 (2.66)	0–10.0

^a^ Standard Deviation.

**Table 4:** Affect regulation measures (PHQ-9, brief COPE, CD-RISC-10) descriptives and baseline, mid-study, and post-study *t*-tests (*n* *=* 91).

Affect regulation measures	Mean	SD^a^	SE^b^	95% CI^c^	*t*-test	*df* ^d^	*p*-value^e^
Patient Health Questionnaire-9 (PHQ-9)							
Baseline	7.28	5.11	1.14				
Mid-study	6.66	4.88	0.51				
Post-study	6.96	5.13	0.54				
Baseline to mid-study	1.63^f^	10.21	0.37	−.12 ± 1.37	1.66	89	.100
Mid-study to post-study	-0.30^f^	3.91	0.41	−1.11 ± .52	−.72	90	.472
Baseline to post-study	1.33^f^	10.89	0.42	−.57 ± 1.10	.64	89	.527
Brief COPE Problem-Focused Coping							
Baseline	21.37	4.53	0.48				
Mid-study	21.51	5.23	0.55				
Post-study	21.92	5.50	0.58				
Baseline to mid-study	−0.13^f^	4.43	0.46	−1.06 ± .79	−0.28	90	.777
Mid-study to post-study	−0.1^f^	4.57	0.48	−1.06 ± .84	−0.23	90	.819
Baseline to post-study	−0.24^f^	4.90	0.51	−1.26 ± .78	−0.47	90	.639
Brief COPE Emotion-Focused Coping							
Baseline	27.97	4.46	0.47				
Mid-study	27.44	5.25	0.55				
Post-study	27.35	5.66	0.59				
Baseline to mid-study	0.53^f^	4.80	0.50	−.47 ± 1.53	1.05	90	.297
Mid-study to post-study	0.09^f^	4.07	0.43	−.76 ± .94	0.21	90	.837
Baseline to post-study	0.62^f^	4.83	0.51	−.39 ± 1.62	1.22	90	.228
Brief COPE Avoidant Coping							
Baseline	14.06	3.16	0.33				
Mid-study	13.57	3.42	0.36				
Post-study	13.76	3.35	0.35				
Baseline to mid-study	0.49^f^	3.35	0.33	−20 ± 1.19	1.41	90	.163
Mid-study to post-study	−0.19^f^	3.10	0.36	−.83 ± .46	−0.58	90	.567
Baseline to post-study	0.31^f^	3.44	0.35	−.41 ± 1.02	0.85	90	.396
Connor Davidson Resilience Scale (CD-RISC 10)							
Baseline	25.89	6.12	0.64				
Mid-study	26.47	5.75	0.60				
Post-study	26.55	6.16	0.65				
Baseline to mid-study	−0.58^f^	3.92	0.41	−1.40 ± .23	−1.42	90	.160
Mid-study to post-study	−0.08^f^	3.89	0.41	−.89 ± .73	−0.19	90	.851
Baseline to post-study	−0.66^f^	4.74	0.50	−1.65 ± .33	−1.33	90	.188

^a^ Standard deviation.

^b^ Standard error.

^c^ Confidence interval of the difference.

^d^ Degrees of freedom.

^e^ We used an alpha level of.05, two-tailed.

^f^ Mean difference.

### 3.2. Preliminary analyses

Our preliminary analyses focused on two research questions to explore: (1) whether or not JoyPop™ app use is associated with reduced affect dysregulation, reduced stress response, and increased perceived social support; and (2) whether changes in affect regulation capacity, stress response, and perceived social support of social work students across the three observations is associated with exposure to pre-study traumatogenic event (ACEs). We first ran correlation matrices between response items, subscales, and scales of the measures included in the preliminary conceptual model (Supplementary Materials) along with sociodemographic characteristics of age, gender, and ethnicity to test for significant relationships. In the interest of parsimony, variables that showed no correlation and were not theoretically essential to the conceptual model were not included in the next level of exploratory analyses, e.g., the well-being construct variables (111).

**Figure 4:**
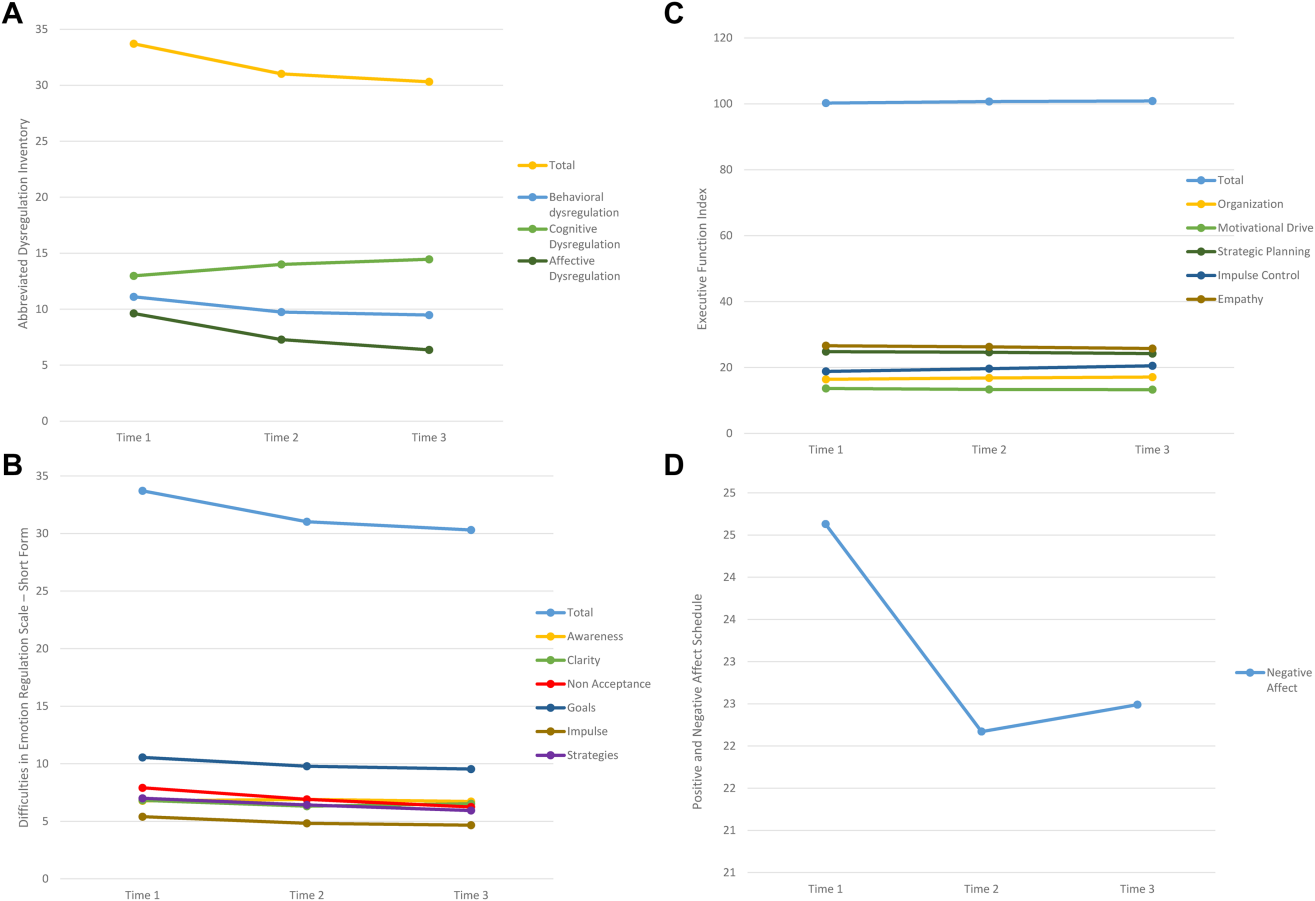
Mean estimates affect regulation measures. (**A**) Abbreviated Dysregulation Inventory. (**B**) Difficulties in Emotion Regulation Scale - Short Form. (**C**) Executive Function Index. (**D**) Positive and Negative Affect Schedule.

**Figure 5:**
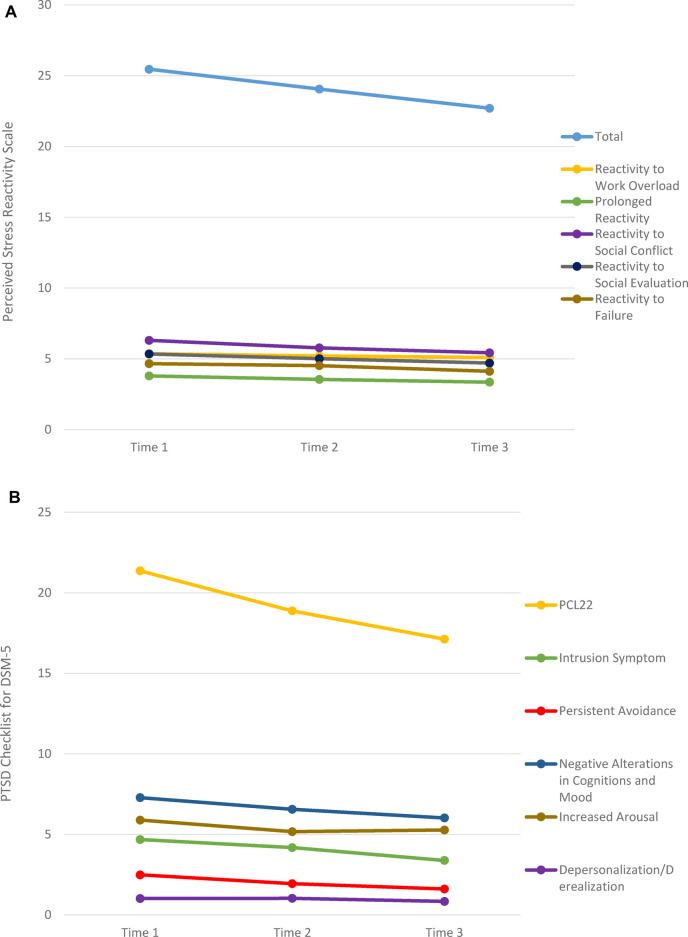
Mean estimates stress response measures. (**A**) Perceived Stress Reactivity Scale. (**B**) PTSD Checklist for DSM-5.

In the second stage of our preliminary analyses, two-tailed paired samples *t*-tests were run on the variables of interest in the respecified model to examine DV change-over-time of the affect regulation capacity, stress responsivity, and perceived social support measures retained in the model (Figure 3 and Tables 3–5). With few exceptions (i.e., PANAS Positive Affect, Brief COPE Emotion-Focused Coping, and PCS), we saw significant mean change across the three time points of data collection in the expected direction (increased affect regulation capacity and social support and decreased stress reactivity, Figures 4, 5). Specifically, several affect regulation measures showed post-app use significant change compared to baseline scores: ADI (pre*M* = 33.54, *SD* = 11.56; post*M* = 30.22, *SD* = 12.46), *t*_(91)_ = 3.10, *p* = .003; DERS-SF (pre*M* = 44.21, *SD* = 12.28; post*M* = 40.85, *SD* = 12.52); *t*(91) = 5.29, *p* < .001; and PANAS Negative Affect Schedule (pre*M* = 24.49, *SD* = 8.32; post*M* = 22.35, *SD* = 8.31), *t*(91) = 2.68, *p* = .009. Amongst the stress responsivity variables, both the PCL-5 (pre*M* = 21.19, *SD* = 16.08; post*M* = 16.85, *SD* = 15.62), *t*(90) = 2.92, *p* = .004 and the PSRS (pre*M* = 25.46, *SD* = 6.96; post*M* = 22.70, *SD* = 7.79), *t*(91) = 4.93, *p* < .001, demonstrated significant change from baseline to the completion of the pilot study period. Both of the two social support measures (Table 5), the MSPSS (pre*M* = 5.52, *SD* = 1.11; post*M* = 5.72, *SD* = 1.09), *t*(91) = −1.79, *p* = .077 and the PCSQ (pre*M* = 43.84, *SD* = 10.47; post*M* = 41.97, *SD* = 12.24), *t*(91) = 1.83, *p* = .070, trended significance in change-over-time. Although not significant in the *t*-tests, several significant correlations emerged on EFI subscales of specific components of cognition, e.g., impulse control and organization. Thus, we included EFI as a DV in the main effects model and testing because of the theoretical salience of the role of executive function in affect regulation and the multiple cognition-focused activities in the JoyPop™ app.

**Table 3:** Affect regulation measures (ADI, DERS-SF, EFI, PANAS) descriptives and baseline, mid-study, and post-study *t*-tests (*n* *=* 91).

Affect regulation measures	Mean	SD^a^	SE^b^	95% CI^c^	*t*-test	*df* ^d^	*p*-value^e^
Abbreviated Dysregulation Inventory (ADI)							
Baseline	33.54	11.56	1.21				
Mid-study	30.95	10.95	1.15				
Post-study	30.22	12.46	1.31				
Baseline to mid-study	2.59^f^	8.19	.86	.89 ± 4.30	3.02	90	.003
Mid-study to post-study	.73^f^	7.92	.83	−.92 ± 2.38	0.87	90	.385
Baseline to post-study	3.32^f^	10.20	1.07	1.19 ± 5.44	3.10	90	.003
Difficulties in Emotion Regulation Scale—Short Form (DERS-SF)							
Baseline	44.21	12.28	1.29				
Mid-study	40.85	12.52	1.31				
Post-study	39.12	11.99	1.26				
Baseline to mid-study	3.36^f^	8.53	0.89	1.59 ± 5.14	3.76	90	<.001
Mid-study to post-study	1.73^f^	7.25	0.76	.21 ± 3.24	2.27	90	0.26
Baseline to post-study	5.09^f^	9.18	0.96	3.18 ± 7.00	5.29	90	<.001
Executive Functioning Index (EFI)							
Baseline	99.69	10.45	1.10				
Mid-study	100.52	10.11	1.06				
Post-study	100.38	11.29	1.18				
Baseline to mid-study	−0.82^f^	8.43	.88	−2.58 ± .93	−.93	90	.353
Mid-study to post-study	0.13^f^	6.93	.73	−1.31 ± 1.58	.18	90	.856
Baseline to post-study	−0.69^f^	9.85	1.03	−2.74 ± 1.36	−.67	90	.504
PANAS Positive Affect Schedule							
Baseline	31.38	7.28	0.76				
Mid-study	30.48	7.66	0.80				
Post-study	30.51	7.68	0.80				
Baseline to mid-study	0.90^f^	6.27	.66	−.40 ± 2.21	1.37	90	.174
Mid-study to post-study	−0.02^f^	6.80	.71	−1.44 ± 1.39	−.03	90	.975
Baseline to post-study	0.88^f^	7.95	.83	−.78 ± 2.54	1.06	90	.294
PANAS Negative Affect Schedule							
Baseline	24.49	8.32	0.87				
Mid-study	22.10	7.75	0.81				
Post-study	22.35	8.31	0.87				
Baseline to mid-study	2.40^f^	7.00	0.73	.94 ± 3.85	3.27	90	.002
Mid-study to post-study	−0.25^f^	5.37	0.56	−1.37 ± .87	−.45	90	.655
Baseline to post-study	2.14^f^	7.64	0.80	.55 ± 3.73	2.68	90	.009

^a^ Standard deviation.

^b^ Standard error.

^c^ Confidence interval of the difference.

^d^ Degrees of freedom.

^e^ We used an alpha level of.05, two-tailed.

^f^ Mean difference.

**Table 5:** Stress responsivity and social support measures means and baseline, mid-study, and post-study *t*-tests (*n* = 91).

Stress responsivity measures	Mean	SD^a^	SE^b^	95% CI^c^	*t*-test	*df* ^d^	*p*-value^e^
PTSD Checklist for DSM-5 (PCL-5)							
Baseline	21.19	16.08	1.71				
Mid-study	18.43	15.92	1.70				
Post-study	16.85	15.62	1.66				
Baseline to mid-study	2.76^f^	12.38	1.32	.14 ± 5.38	2.09	87	.039
Mid-study to post-study	1.87^f^	10.93	1.16	−.44 ± 4.17	1.61	88	.111
Baseline to post-study	4.70^f^	15.27	1.61	1.50 ± 7.90	2.92	89	.004
Perceived Stress Reactivity Scale (PSRS)							
Baseline	25.46	6.96	0.73				
Mid-study	24.05	7.43	0.78				
Post-study	22.70	7.79	0.82				
Baseline to mid-study	1.41^f^	4.28	0.45	.52 ± 2.30	3.14	90	.002
Mid-study to post-study	1.35^f^	4.35	0.46	.45 ± 2.26	2.97	90	.004
Baseline to post-study	2.76^f^	5.34	0.56	1.64 ± 3.87	4.93	90	<.001
Multidimensional Scale of Perceived Social Support (MSPSS)							
Baseline	5.52	1.11	0.12				
Mid-study	5.56	1.12	0.12				
Post-study	5.72	1.09	0.11				
Baseline to mid-study	−0.04^f^	1.19	0.12	−.29 ± .21	−.32	90	.749
Mid-study to post-study	−0.16^f^	1.04	0.11	−37 ± .06	−1.44	90	.154
Baseline to post-study	−0.20^f^	1.05	0.11	−.41 ± .021	−1.79	90	.077
Perceived Community Support Questionnaire (PCSQ)							
Baseline	43.84	10.47	1.11				
Mid-study	43.38	10.78	1.14				
Post-study	41.97	12.24	1.30				
Baseline to mid-study	0.46^f^	9.95	1.05	−1.63 ± 2.56	.44	88	.663
Mid-study to post-study	1.45^f^	7.28	0.78	−09 ± 3.00	1.87	87	.064
Baseline to post-study	2.16^f^	11.17	1.18	−.18 ± 4.49	1.83	89	.070

^a^ Standard deviation.

^b^ Standard error.

^c^ Confidence interval of the difference.

^d^ Degrees of freedom.

^e^ We used an alpha level of.05, two-tailed.

^f^ Mean difference.

Before conducting the main analysis, we explored the correlations of baseline traumatogenic exposure with the ACEQ, CTQ, and LEC-5 as IVs in the model in relation to the entire scale and significant subscale post-app measures identified in the *t*-tests. No significant IV correlations were present in the matrix of DVs, apart from the ACEQ. To further explore the possibility that increased exposure through frequency or chronicity might have additional interaction with the DVs, we created indicator variables to explore aggregate frequency of exposure with the binary 11-item ACEQ scale categorized by exposure counts: zero ACEs, 1–4 ACEs, 5+ ACEs. To explore chronicity, we created a categorical variable to capture the response option that we had added with which respondents indicated that they had experienced an ACEQ item multiple times. This measure was scored 0 for “never”, 1 for “at least once”, and 2 for “many times.” Based on the results of the *t*-tests and further correlation exploration with the additional ACEs variables, we again respecified to determine the final model shown in Figure 3.

### 3.3. Main analysis

To further explore the two resilience-focused research questions, we ran several GEE models with the imputed and restructured dataset. First, in the absence of sufficient variation in the DAU variable, change-over-time on affect regulation, stress reactivity, and social support was tested using the TIME indicator variable (created in the restructuring) as the IV to test the autoregressive relationship of marginal change with each of the eight DVs included in the final model (Figure 3). Secondly, we explored the relationship between pre-study vulnerabilities represented by the five ACEQ variables of traumatogenic exposure as IVs that might influence change-over-time associated with JoyPop™ app use with each of the eight DVs included in the final model. Although GEE does not tolerate complex modeling, it is possible to test multiple IVs, covariates, and interaction effects with one DV per equation. Thus, our third analysis tested for change-over-time, ACEs, and their interaction effects on the eight DVs of the final model.

We first explored the relationship between the indicator variable of TIME and eight DVs. The first three rows of Tables 6–8 contain the pooled marginal mean estimates of the imputed dataset for all final model DVs (Figure 3), which varied little from the non-imputed conditional means (see Tables 3, 4). Across all three time points, there was consistent change in reducing affect dysregulation and stress reactivity and small increases in perceived social support. In general, the largest amount of change in DVs happened in the first two weeks of app use between the administration of the pre-study measures and the mid-study measures, which were completed within 7 days following the mid-point (14 days) of the 28-day observation period. The rate of change is reported in rows 4–6 in Tables 6–9, which contain the coefficient scores of the DVs [mean difference (*I*-*J*)] with their standard errors, Wald *Χ*^2^ 95% confidence intervals, *p* values, and the test of model effects statistics. In addition to change-over-time occurring in the expected direction (i.e., reduced affect dysregulation and stress reactivity and increased social support), most of the measure changes were significant at both the scale and subscales levels.

**Table 6:** GEE estimated marginal means and coefficients for time and affect regulation measures (ADI, PANAS) baseline, mid-study, and post-study^a^ (*n* = 91).

Time	Mean	*β*	SE^b^	95% Wald CI^c^	Wald *χ*^2^	*df* ^d^	*p-*value^e^	Model Effects		
								Wald χ^2^	*df* ^d^	*p-*value^e^
Abbreviated Dysregulation Inventory (ADI)										
Baseline	33.69	1.20	31.35 ± 36.04							
Mid-study	31.04	1.14	28.80 ± 33.28							
Post-study	30.32	1.29	27.79 ± 32.84							
Intercept		33.69	1.20	31.35 ± 36.04	790.67	1	<.001	847.18	1	<.001
Baseline to mid-study		−2.65	0.86	−4.35 ± −1.29	9.98	1	.002	11.78^f^	2	.003
Baseline to post-study		−3.38	1.07	−5.47 ± −0.96	9.41	1	.002			
ADI behavioral dysregulation subscale										
Baseline	11.10	0.55	10.02 ± 12.18							
Mid-study	9.74		0.57	8.62 ± 10.85						
Post-study	9.47		0.61	8.27 ± 10.67						
Intercept		11.10	0.55	10.02 ± 12.18	405.75	1	<.001	358.05	1	<.001
Baseline to mid-study		−1.36	0.36	−2.06 ± −.66	14.63	1	<.001	19.03^f^	2	<.001
Baseline to post-study		−1.63	0.43	−2.47 ± −.80	14.54	1	<.001			
ADI cognitive regulation subscale										
Baseline	12.98	0.52	11.96 ± 14.00							
Mid-study	14.00	0.58	12.86 ± 15.14							
Post-study	14.48	0.62	13.27 ± 15.69							
Intercept		12.98	0.52	11.96 ± 14.00	624.52	1	<.001	834.75	1	<.001
Baseline to mid-study		1.02	0.50	0.05 ± 1.99	4.22	1	.040	8.15^f^	2	.017
Baseline to post-study		1.50	0.57	0.38 ± 2.61	6.93	1	.009			
ADI affective dysregulation subscale										
Baseline	9.61		0.60	8.42 ± 10.79						
Mid-study	7.30		0.49	6.34 ± 8.26						
Post-study	6.37		0.54	5.31 ± 7.43						
Intercept		9.61	0.61	8.42 ± 10.80	251.13	1	<.001	256.00	1	<.001
Baseline to mid-study		−2.31	0.47	−3.23 ± −1.39	24.32	1	<.001	41.13^f^	2	<.001
Baseline to post-study		−3.24	0.51	−4.23 ± −2.25	41.13	1	<.001			
PANAS Negative Affect Schedule										
Baseline	24.61		0.87	22.91 ± 26.32						
Mid-study	22.18		0.80	20.61 ± 23.74						
Post-study	22.48		0.85	20.81 ± 24.16						
Intercept		24.61	0.87	22.91 ± 26.32	800.91	1	<.001	953.79	1	<.001
Baseline to mid-study		−2.44	0.72	−3.84 ± −1.03	11.40	1	.001	11.53^f^	2	.003
Baseline to post-study		−2.13	0.78	−3.66 ± −0.60	7.40	1	.007			

^a^ Pooled results of the imputed dataset.

^b^ Standard error.

^c^ Confidence interval of the difference.

^d^ Degrees of freedom.

^e^ Significant at the.05 level.

^f^ Test of model effects values for Time.

**Table 7:** GEE estimated marginal means and coefficients for time and affect regulation measures (DERS-SF) baseline, mid-study, and post-study^a^ (*n* = 91).

Time	Mean	*β*	SE^b^	95% Wald CI^c^	Wald χ^2^	*df* ^d^	*p-*value^d^	Model Effects		
								Wald χ^2^	*df* ^d^	*p-*value^e^
Difficulties in Emotion Regulation Scale—Short Form (DERS-SF)										
Baseline	44.49	1.27	41.99 ± 46.99							
Mid-study	41.13	1.28	38.62 ± 43.63							
Post-study	39.59	1.25	37.14 ± 42.05							
Intercept		44.49	1.28	41.99 ± 46.99	1,213.77	1	<.001	1,245.18	1	<.001
Baseline to mid-study		−3.37	0.83	−4.99 ± −1.74	16.19	1	<.001	28.02^f^	2	<.001
Baseline to post-study		−4.90	0.93	−6.73 ± −3.07	27.84	1	<.001			
DERS-SF awareness subscale										
Baseline	6.78		0.24	6.31 ± 7.26						
Mid-study	6.90		0.24	6.44 ± 7.36						
Post-study	6.72		0.24	6.25 ± 7.19						
Intercept		6.79	0.25	6.30 ± 7.27	754.48	1	<.001	1,130.78	1	<.001
Baseline to mid-study		0.12	0.25	−0.37 ± .61	0.24	1	.628	1.19^f^	2	.569
Baseline to post-study		−0.07	0.24	−0.54 ± .41	0.13	1	.746			
DERS-SF clarity subscale										
Baseline	6.83		0.26	6.32 ± 7.34						
Mid-study	6.31		0.28	5.76 ± 6.85						
Post-study	6.52		0.28	5.96 ± 7.07						
Intercept		6.83	0.26	6.32 ± 7.34	678.20	1	<.001	733.17	1	<.001
Baseline to mid-study		−0.52	0.23	−0.98 ± −0.07	4.88	1	.028	4.89^f^	2	.089
Baseline to post-study		−0.31	0.24	−0.77 ± −0.15	1.71	1	.193			
DERS-SF nonacceptance subscale										
Baseline	7.91		0.42	7.09 ± 8.72						
Mid-study	6.91		0.39	6.14 ± 7.67						
Post-study	6.23		0.35	5.55 ± 6.91						
Intercept		7.91	0.42	7.09 ± 8.72	358.15	1	<.001	394.52	1	<.001
Baseline to mid-study		−1.00	0.26	−1.51 ± −0.49	15.23	1	<.001	30.74^f^	2	<.001
Baseline to post-study		−1.67	0.30	−2.27 ± −1.08	30.41	1	<.001			
DERS-SF goals subscale										
Baseline	10.55	0.33	9.90 ± 11.21							
Mid-study	9.78		0.37	9.06 ± 10.50						
Post-study	9.56		0.34	8.88 ± 10.23						
Intercept		10.55	0.34	9.90 ± 11.21	989.20	1	<.001	1,045.04	1	<.001
Baseline to mid-study		−0.77	0.31	−1.38 ± −0.16	5.93	1	.015	10.23^f^	2	.006
Baseline to post-study		−1.00	0.32	−1.63 ± −0.37	9.92	1	.002			
DERS-SF impulse subscale										
Baseline	5.42		0.31	4.82 ± 6.02						
Mid-study	4.81		0.23	4.35 ± 5.27						
Post-study	4.65		0.24	4.17 ± 5.13						
Intercept		5.42	0.31	4.81 ± 6.03	303.66	1	<.001	502.56	1	<.001
Baseline to mid-study		−0.61	0.27	−1.15 ± −0.08	5.04	1	.025	6.64^f^	2	.039
Baseline to post-study		−0.77	0.30	−1.37 ± −0.18	6.56	1	.012			
DERS-SF strategies subscale										
Baseline	7.00		0.34	6.34 ± 7.67						
Mid-study	6.42		0.34	5.76 ± 7.08						
Post-study	5.92		0.31	5.31 ± 6.54						
Intercept		7.00	0.34	6.34 ± 7.67	423.31	1	<.001	435.43	1	<.001
Baseline to mid-study		−0.58	0.22	−1.00 ± −0.16	7.35	1	.007	22.41^f^	2	<.001
Baseline to post-study		−1.08	0.23	−1.53 ± −0.63	21.68	1	<.001			

^a^ Pooled results of the imputed dataset.

^b^ Standard error.

^c^ Confidence interval of the difference.

^d^ Degrees of freedom.

^e^ Significant at the.05 level.

^f^ Test of model effects values for Time.

**Table 8:** GEE estimated marginal means and coefficients for time and affect regulation measures (EFI) baseline, mid-study, and post-study^a^ (*n* = 91).

Time	Mean	*β*	SE^b^	95% Wald CI^c^	Wald χ^2^	*df* ^d^				
								Wald χ^2^	*df* ^d^	*p-*value^e^
Executive Functioning Index (EFI)										
Baseline	100.25	0.99	98.31 ± 102.19							
Mid-study	100.69	1.05	98.63 ± 102.75							
Post-study	100.85	1.14	98.63 ± 103.08							
Intercept		100.25	1.00	98.30 ± 102.20	9,824.23	1	<.001	10,824.77	1	<.001
Baseline to mid-study		0.45	0.77	−1.07 ± 1.96	0.43	1	.528	0.54^f^	2	.765
Baseline to post-study		0.60	0.91	−1.18 ± 2.39	0.44	1	.506			
EFI organization subscale										
Baseline	16.41		0.41	15.61 ± 17.21						
Mid-study	16.84		0.38	16.09 ± 17.59						
Post-study	17.10		0.40	16.32 ± 17.88						
Intercept		16.41	0.41	15.60 ± 17.21	1,596.51	1	<.001	2,320.54	1	<.001
Baseline to mid-study		0.43	0.33	−0.22 ± 1.09	1.68	1	.200	4.02^f^	2	.137
Baseline to post-study		0.69	0.35	0.00 ± 1.38	3.96	1	.048			
EFI motivational drive subscale										
Baseline	13.63		0.28	13.07 ± 14.18						
Mid-study	13.34		0.31	12.74 ± 13.94						
Post-study	13.27		0.32	12.64 ± 13.91						
Intercept		13.63	0.28	13.07 ± 14.18	2,338.26	1	<.001	2,608.26	1	<.001
Baseline to mid-study		−0.29	0.26	−0.80 ± 0.23	1.15	1	.285	1.58^f^	2	.455
Baseline to post-study		−0.35	0.29	−0.93 ± 0.23	1.38	1	.240			
EFI strategic planning subscale										
Baseline	24.81	0.42	23.99 ± 25.6.							
Mid-study	24.60	0.41	23.79 ± 25.41							
Post-study	24.19	0.46	23.28 ± 25.10							
Intercept		24.81	0.42	23.99 ± 25.63	3,448.46	1	<.001	4,185.03	1	<.001
Baseline to mid-study		−0.21	0.35	−0.89 ± 0.47	0.34	1	.577	2.32^f^	2	.315
Baseline to post-study		−0.62	0.42	−1.44 ± 0.21	2.07	1	.154			
EFI impulse control subscale										
Baseline	18.82	0.31	18.22 ± 19.42							
Mid-study	19.66	0.28	19.11 ± 20.21							
Post-study	20.53	0.27	20.00 ± 21.06							
Intercept		18.82	0.31	18.20 ± 19.43	3,585.19	1	<.001	6,111.42	1	<.001
Baseline to mid-study		0.84	0.25	0.35 ± 1.33	11.62	1	.001	37.33^f^	2	<.001
Baseline to post-study		1.71	0.28	1.16 ± 2.27	36.62	1	<.001			
EFI empathy subscale										
Baseline	26.58	0.34	25.91 ± 27.26							
Mid-study	26.25	0.33	25.60 ± 26.91							
Post-study	25.76	0.34	25.10 ± 26.42							
Intercept		26.59	0.34	25.91 ± 27.26	5,841.00	1	<.001	7,816.76	1	<.001
Baseline to mid-study		−0.33	0.30	−0.93 ± 0.26	1.03	1	.347	7.07^f^	2	.032
Baseline to post-study		−0.83	0.34	−1.49 ± −0.17	6.04	1	.014			
Baseline to post-study		−2.13	0.78	−3.66 ± −0.60	7.40	1	.007			

^a^ Pooled results of the imputed dataset.

^b^ Standard error.

^c^ Confidence interval of the difference.

^d^ Degrees of freedom.

^e^ Significant at the.05 level.

^f^ Test of model effects values for Time.

**Table 9:** GEE estimated marginal means and coefficients for time and stress responsivity measure baseline, mid-study, and post-study^a^ (*n* = 91).

Time	Mean	*β*	SE^b^	95% Wald CI^c^	Wald χ^2^	*df* ^V^	*p-*value^d^	Model Effects		
								Wald χ^2^	*df* ^d^	*p-*value^e^
Perceived Stress Reactivity Scale (PSRS)										
Baseline	25.51		0.69	24.16 ± 26.87						
Mid-study	24.06		0.71	22.66 ± 25.46						
Post-study	22.87		0.77	21.36 ± 24.37						
Intercept		25.51	0.72	24.11 ± 26.92	1,265.62	1	<.001	1,144.78	1	<.001
Baseline to mid-study		−1.45	0.45	−2.33 ± −0.57	10.38	1	.001	23.29^f^	2	<.001
Baseline to post-study		−2.65	0.55	−3.73 ± −1.56	23.23	1	<.001			
PSRS work overload subscale										
Baseline	5.38		0.24	4.92 ± 5.85						
Mid-study	5.22		0.24	4.74 ± 5.69						
Post-study	5.12		0.25	4.62 ± 5.62						
Intercept		5.38	0.24	4.91 ± 5.85	501.66	1	<.001	513.86	1	<.001
Baseline to mid-study		−0.17	0.17	−0.51 ± 0.18	0.90	1	.345	1.84^f^	2	.398
Baseline to post-study		−0.26	0.19	−0.64 ± 0.12	1.81	1	.179			
PSRS prolonged reactivity subscale										
Baseline	3.81		0.19	3.44 ± 4.18						
Mid-study	3.55		0.19	3.17 ± 3.93						
Post-study	3.38		0.21	2.97 ± 3.79						
Intercept		3.81	0.19	3.44 ± 4.18	400.01	1	<.001	409.61	1	<.001
Baseline to mid-study		−0.26	0.15	−0.56 ± 0.04	2.79	1	.096	5.94^f^	2	.053
Baseline to post-study		−0.43	0.18	−0.78 ± −0.08	5.92	1	.016			
PSRS social conflict subscale										
Baseline	6.31		0.20	5.93 ± 6.70						
Mid-study	5.77		0.21	5.36 ± 6.18						
Post-study	5.47		0.22	5.04 ± 5.90						
Intercept		6.31	0.20	5.92 ± 6.71	995.57	1	<.001	960.77	1	<.001
Baseline to mid-study		−0.55	0.18	−0.89 ± −0.46	9.59	1	.002	19.53^f^	2	<.001
Baseline to post-study		−0.84	0.19	−1.23 ± −0.20	19.24	1	<.001			
PSRS social evaluation subscale										
Baseline	5.34		0.21	4.92 ± 5.76						
Mid-study	5.01		0.20	4.61 ± 5.41						
Post-study	4.74		0.22	4.31 ± 5.16						
Intercept		5.34	0.22	4.90 ± 5.78	572.68	1	<.001	632.30	1	<.001
Baseline to mid-study		−0.33	0.16	−0.64 ± −0.02	4.45	1	.035	9.26^f^	2	.010
Baseline to post-study		−0.60	0.20	−1.00 ± −0.21	9.12	1	.003			
PSRS reactivity to failure subscale										
Baseline	4.67		0.15	4.37 ± 4.97						
Mid-study	4.52		0.15	4.21 ± 4.82						
Post-study	4.16		0.16	3.84 ± 4.47						
Intercept		4.67	0.15	4.37 ± 4.97	914.49	1	<.001	1,040.31	1	<.001
Baseline to mid-study		−0.15	0.14	−0.42 ± 0.12	1.18	1	.279	15.02^f^	2	.001
Baseline to post-study		−0.51	0.14	−0.78 ± −0.24	14.19	1	<.001			

^a^ Pooled results of the imputed dataset.

^b^ Standard error.

^c^ Confidence interval of the difference.

^d^ Degrees of freedom.

^e^ Significant at the.05 level.

^f^ Test of model effects values for Time.

For example, within the ADI scale, we see a pre- to post-study decrease in the total scale (*β* = −3.38, *p* *=* <.001), the subscales of behavioral (*β* = −1.63, *p* *=* <.001) and affect dysregulation (*β* = −3.24, *p* *=* <.001), and an increase in the reverse-scored cognitive regulation subscale (*β* = 1.50, *p* *=* .009), as expected (Table 6). Similarly, the DERS-SF, a very popular measure of emotion regulation (93), showed significant decreases over time of the total scale and all subscales except the reverse-scored awareness subscale and pre- to post-study change in clarity (Table 7). In contrast, the EFI full measure marginal mean differences reflected expected but non-significant increases over both time points (Table 8). Nonetheless, several subscales were significant pre- to post-study, notably organization (*β* = 0.69, *p* *=* .048) and impulse control (*β* = 1.71, *p* *=* <.001). However, empathy decreased (*β* = −0.83, *p* *=* .014). The PANAS measure showed a pre- to post-study reduction in negative affect (*β* = −2.13, *p* *=* .007) at both observation points (Table 6).

When testing change-over-time of stress responsivity, in addition to significant reduction pre- to mid-study (*β* = −1.45, *p* *=* <.001) and pre- to post-study (*β* = −2.65, *p* *=* <.001) for the full scale, we found that all subscales, apart from work overload, showed small but significant reductions (Table 9). The PCL-5 full measure of PTSD showed a reduction from pre- to post-study (*β* = −4.24, *p* *=* .006) and three out of the five subscales (Supplementary Table S1). Results of the change-over-time test of the MSPSS full scale trended significance and a small significant increase in perceived support from the respondents' significant other from pre- to post-study (*β* = 0.30, *p* *=* .038).

We then tested change-over-time in the eight DVs with the five ACE variables of traumatogenic pre-study exposure (i.e., ACEQ, ACEchron, ACEzero, ACE1–4, ACE5+) as covariates (Table 10). The marginal model results showed considerable variation across measures and within measures by subscales. Only one of the full scales showed any significant change, and there was no perceptible pattern in the very few significant subscales results. We nonetheless ran a final model with both TIME and ACEs variables as covariates and added an interaction term of TIME by each of the ACE variables. As expected based on the previous results, there were very few significant interaction effects. However, the results shown in Table 10 of the stress responsivity measure (PSRS) tested with each of the 5 ACEs covariates and the indicator variable of TIME show the marginal variance in change-over-time in the different subpopulations delineated by the different groupings of rate and count of pre-study traumatogenic exposure. In addition to TIME, several of the ACE variables accounted for variance in decreased perceived stress by increased summed exposure (ACEQ *β* = −0.673, *p* *=* .025), increased chronicity (ACEchron *β* = −0.378, *p* *=* .031), exposure to more than 5 reported ACEs (ACE5+ *β* = −3.786, *p* *=* .030). Similar reduction was present in the social evaluation subscale for ACEQ (*β* = −0.673, *p* *=* .025), ACEchron (*β* = −0.156, *p* *=* .002), and ACE5+ (*β* = −1.538, *p* *=* .002; Supplementary Table S2). In both the total scale and subscale, the ACE5+ indicator subpopulation saw a substantially greater reduction in perceived stress than the other groups. Conversely, the ACE1–4 group saw an increase in perceived stress (*β* = 1.150, *p* *=* .009) in the social evaluation subscale results. There were several mid-study x ACEs interaction effects, though there did not seem to be a perceptible pattern.

**Table 10:** GEE estimated marginal means and coefficients for PSRS, time, and time x ACEs baseline, mid-study, and post-study^a^ (*n* = 91).

Time	β	SE^b^	95% Wald CI^c^	Wald *Χ*^2^	*df* ^d^	*p*-value^e^	Model effects		
							Wald *Χ*^2^	*df* ^d^	*p*-value^e^
Perceived Stress Reactivity Scale (PSRS)									
Intercept	27.632	1.05	25.57 ± 29.69	693.29	1	<.001	723.80	1	<.001
ACEQ^i^	−0.673	0.30	−1.26 ± −0.09	5.05	1	.025	8.59^f^	1	.003
Mid-study	0.015	0.73	−1.42 ± 1.45	0.00	1	.984	8.17^g^	2	.017
Post-study	−1.945	0.92	−3.75 ± −0.15	4.52	1	.034	6.44^h^	2	.041
Mid-study x ACEQ	−0.467	0.20	−0.86 ± −0.08	5.52	1	.019			
Post-study x ACEQ	−0.223	0.24	−0.70 ± 0.25	0.91	1	.359			
Intercept	27.140	0.95	25.27 ± 29.01	811.11	1	<.001	873.44	1	<.001
ACEchron^j^	−0.378	0.17	−0.72 ± −0.04	4.66	1	.031	8.84^f^	1	.003
Mid-study	−0.364	0.71	−1.76 ± 1.03	0.26	1	.608	6.50^g^	2	.039
Post-study	−1.926	0.86	−3.62 ± −0.24	4.98	1	.025	3.19^h^	2	.203
Mid-study x ACEchron	−0.253	0.14	−0.54 ± 0.03	3.06	1	.080			
Post-study x ACEchron	−0.167	0.16	−0.48 ± 0.14	1.29	1	.286			
Intercept	25.196	0.78	23.66 ± 26.73	1,027.41	1	<.001	915.42	1	<.001
ACEzero^k^	1.881	1.88	−1.81 ± 5.57	1.03	1	.317	2.92^f^	1	.088
Mid-study	−1.915	0.47	−2.83 ± −1.00	16.79	1	<.001	25.31^g^	2	<.001
Post-study	−2.777	0.59	−3.93 ± −1.63	22.89	1	<.001	7.06^h^	2	.033
Mid-study x ACEzero	2.734	1.27	0.24 ± 5.23	4.73	1	.032			
Post-study x ACEzero	0.786	1.71	−2.56 ± 4.14	0.24	1	.645			
Intercept	24.386	1.23	21.98 ± 26.79	394.38	1	<.001	337.95	1	<.001
ACE1–4^l^	2.112	1.46	−0.74 ± 4.96	2.12	1	.147	3.49^f^	1	.062
Mid-study	−1.614	0.76	−3.11 ± −0.12	4.39	1	.034	17.22^g^	2	<.001
Post-study	−3.391	0.86	−5.09 ± −1.70	15.97	1	<.001	2.11^h^	2	.370
Mid-study x ACE1–4	0.301	0.92	−1.51 ± 2.11	0.10	1	.745			
Post-study x ACE1–4	1.396	1.13	−0.81 ± 3.61	1.68	1	.216			
Intercept	26.638	0.72	25.23 ± 28.05	1,365.29	1	<.001	1,388.08	1	<.001
ACE5+^m^	−3.786	1.74	−7.20 ± −0.37	4.74	1	.030	9.71^f^	1	.002
Mid-study	−0.800	0.50	−1.77 ± 0.17	2.60	1	.107	9.49^g^	2	.009
Post-study	−1.997	0.65	−3.28 ± −0.72	9.51	1	.002	5.04^h^	2	.081
Mid-study x ACE5+	−2.200	1.01	−4.19 ± −0.21	4.62	1	.030			
Post-study x ACE5+	−2.188	1.18	−4.50 ± 0.12	3.52	1	.063			

^a^Pooled results of the imputed dataset.

^b^Standard error.

^c^Confidence interval of the difference.

^d^Degrees of freedom.

^e^We used an alpha level of .05.

^f^Test of model effects values for ACEs.

^g^Test of model effects values for time.

^h^Test of model effects values for time × ACEs.

^i^Dichotomous 11-item self-report measure summed.

^j^Chronicity of exposure 11-item self-report measure summed.

^k^Indicator variable of zero ACEs reported on ACEQ.

^lI^ndicator variable of 1–4 ACEs reported on ACEQ.

^m^Indicator variable of five or more ACEs reported on ACEQ.

## 4. Discussion

As mental health and resilience apps continue to flood the market, it is critical that users are provided evidence supporting which apps may be effective for what, for whom, and how. Our pilot study of the JoyPop™ app sought to evaluate whether or not there were any changes based on app use in outcomes related to three core constructs of resilience: affect regulation capacity, stress responsivity, and social support. We tested the app with social work trainees, most of whom were in their second year of university, enrolled in a BSW program, and older youth [under 30 years old, (117)]. The preliminary analysis identified significant change-over-time in four affect regulation measures, three stress responsivity measures, and one social support measure. The results of the GEE modeling indicated that the autoregressive relationship of marginal change-over-time associated with JoyPop™ app use consistently reduced affect dysregulation, measured by the ADI, DERS, and PANAS Negative Affect scale and most of their subscales. Unexpectedly, measures of positive affect, resilience, coping, depression, and most subscales of executive function showed no significant change associated with app use. Both perceived stress and PTSD levels were significantly lower over time, along with minimal increases in perceived social support. Most substantive change occurred in the first 2 weeks of app use. Given the comparatively high rate of self-reported traumatogenic exposure in the study sample, we tested the association between any, chronic, and frequent ACE exposure and the measures of affect regulation, stress responsivity, and social support. Significant associations were few and inconsistent. Nonetheless, the exploratory findings from our pilot study suggest that consistent use of the app may enhance multidimensional resilience amongst university students reporting multiple traumatogenic exposures. Our findings support an approach modeling resilience as a complex, dynamic, and multicomponent process supported by resources within and between individuals (1, 3, 52, 56, 68, 118).

Resilience is by definition a process of change in response to adversity. Consequently, research on resilience interventions optimally accounts for: (1) exposure to high stress (i.e., traumatogenic experiences) that precipitates change in biopsychosocial functioning and behavior; (2) identification of the mechanisms and processes that underlie change processes; and (3) critical evaluation of intervention outcomes accounting for complexity and dynamic interaction of change processes and predisposing factors. The JoyPop™ app was designed to enhance resilience for vulnerable youth (12–14), thus we assessed baseline adversity measured by three scales: ACEQ, CTQ, and LEC-5. In the absence of significant correlations between any DVs and the CTQ and the LEC-5 item and scale scores, we focused on the ACEQ as the measure of precipitating traumatogenic exposure for our analyses. The ACEQ has been used widely since the publication of the first findings claiming association between childhood traumatogenic exposure and adult health outcomes [e.g., (18, 23)].

However, use of the ACEQ beyond its original epidemiologic scope has come under criticism [see (21, 27, 30, 31)]. Three main concerns regarding ACEs research findings are the conflation of traumatogenic exposure with effect, inferred causality based on retrospective cross-sectional self-report ACEQ data (such as ours), and the lack of nuance in ACEQ data regarding the scope of exposure (i.e., frequency, severity, and chronicity) to the events included in the questionnaire (30, 31, 119). In our analysis, we sought to add nuance to the measure by including a question asking whether or not an event, if experienced at least once, was also experienced multiple times (i.e., chronicity). The results demonstrated some support for this approach. Our student sample reported higher than average rates of ACEs with 30% of the participants reporting more than four ACEs and over half of the sample reporting some degree of chronic exposure. Elevated rates of traumatogenic event exposure are not uncommon, however, in samples comprised of persons in or training to join helping professions, many of whom have lived experiences of the same biopsychosocial vulnerabilities as service seeking populations (78–80). Thus, the heightened baseline vulnerability of our sample suggests relevance of our pilot findings for the target group for the JoyPop™ app, i.e., youth who have experienced high adversity.

Nonetheless, even with our efforts to capture more nuance in ACE exposure, we found little predictive value of change in the three components of resilience tested. There are several possible reasons for these results. As mentioned in a similar pilot study with the JoyPop™ app, university students, regardless of their ACE exposure, may already be fairly internally and externally well-resourced and able to respond flexibly to heightened stress (14). Also as noted, ACE exposure does not equal effect nor can a causal relationship be reliably modeled with retrospective self-reported cross-sectional exposure data (27–31). Although the ACEQ included multiple ACE exposures, a measure that specifically tests for polyvictimization including individual and community-level adversity [e.g., (26)] would increase the predictive strength over the several unrelated exposure measures we included [e.g., (19, 30)]. Importantly, also missing from our assessment is testing for instigating adversity (e.g., bullying, dating violence, etc.), which is highly correlated with victimization and high affect dysregulation, signaling possible adaptations to stress that inhibit well-being (120, 121). Indeed, a complex assessment of the interaction of factors which buffer, exacerbate, or protect against enduring effects from traumatogenic exposure is common in child development and trauma-informed intervention research [e.g., (21, 50, 52, 118, 122)].

Grych et al. (118) have developed a conceptual framework—the Resilience Portfolio Model—a strengths-based means to evaluate a more complex and dynamic understanding of the relationship between adversity exposure, stress response adaptations, and resilience for individuals and communities. Many components of the Resilience Portfolio Model (118) and Gross et al.'s (55) affect regulation stages and strategies framework were included in our original conceptual model (Supplementary Materials) including coping, post-traumatic growth, self-empathy, and community support but not retained in the final model due to lack of significance. Future evaluation of the JoyPop™ app intervention could benefit from a more comprehensive assessment of baseline indicators of pre-study traumatogenic exposure and adaptive strategies and behaviors [e.g., (123)] to test the effects of app use.

Change-over-time adaptation to adversity that results in resilience or inhibits well-being in specific environments is driven by repetition (15, 16, 42–45). Thus, repetition is the central mechanism of the JoyPop™ app intervention. In the pilot study, we asked students to use the app twice daily for 4 weeks to create the condition of repetition. Our sample was highly compliant and used the app an average of 26.9 days (*SD* = 1.90) out of 28, likely an artifact of receiving course credit for participation in the study. We tested for change-over-time by comparing baseline scores on DVs to mid-study scores, which were in turn compared to post-study scores. We also compared baseline to post-study scores, given the short time frame between tests. As expected in repeated measures, the autoregressive correlations were strong between the variables in each wave of data. Observed mean change in the expected direction was evident in each DV of the three resilience constructs of affect regulation, stress responsivity, and social support (Figures 4, 5), which is promising.

Furthermore, *t*-tests were significant (Tables 3–5) in three affect measures (ADI, DERS-SF, PANAS Negative Affect Scale) and two stress measures (PCL-5 and PSRS), demonstrating that with consistent use of the app, study participants experienced decreased affect dysregulation and reactivity to stress as hypothesized. Both social support measures trended significance (*p* = <.10), and one (MSPSS) was retained in the respecified model (Figure 3) given that social support is such an integral component of resilience and was so profoundly impacted by the COVID-19 pandemic. Although we saw no significant difference in the sample on social support measures between the half who completed the study prior to COVID-19 pandemic lockdown measures and those who participated during the pandemic, there may still have been an effect.

Following the conditional modeling of change-over-time with individual-level *t*-tests, we further examined the effect of consistent use of the app with the full sample in between-participant comparisons using marginal modeling GEE (112, 113). The JoyPop™ app intervention is comprised of resilience-related features with a strong evidence base (12–14), much of which is grounded in the neuroscience of stress responsivity adaptation research on behavioral change (15, 16, 44, 45). The specific psychophysiological mechanisms of adaptation have been clearly articulated in a comprehensive evidence base of the mechanisms and processes of environmentally adaptive change via repetition [e.g., (15, 16, 45)] to specific conditions, for example during the critical adolescent affect regulation developmental window [e.g., (56–59)], or following exposure to childhood adversity [e.g., (22)]. Repetition is fundamental to generating changes in brain structure and function primarily through the mechanisms of brain plasticity, epigenetics, and allostasis (41–45). Our marginal modeling affirmed that repetitive daily use of the JoyPop™ app features enhanced multiple resilience processes embedded in the app design (Figure 2) and hypothesised in our evaluation.

The results of the GEE analysis exploring marginal change-over-time with total scale measures and their multiple subscales (Tables 6–9) highlight the relevance of testing a conceptual framework grounded in the complexity and interactive dynamics of resilience to better understand the underlying mechanisms and processes of change [e.g., (16, 22, 55, 118)]. Conducting this exploration with marginal modeling affords the opportunity to focus on group change in constructs rather than individual-level change (112, 113). The original conceptual model (see Supplementary Material) included six different measures of affect regulation, three of which showed no significant change-over-time in *t*-tests of GEE modeling. Although 25% of the sample reported baseline clinical-level depressive symptoms (PHQ-9), changes in the rates were not significant at any observation. As well, despite their conceptual salience in the JoyPop™ app intervention design, the lack of significant results for executive function (EFI), resilience (CD-RISC-10), and coping (Brief COPE) was surprising.

One explanation for these results, however, might be a lack of synchronization between the design intent/action of app features and the concept/outcome that is measured in specific instruments in the analysis. Indeed, the wording of scale items in the EFI, CD-RISC-10, and Brief COPE are more oriented to fixed traits, whereas app feature use induces states of affect regulation (i.e., diaphragmatic breathing). For example, an EFI (94) item states “I only have to make a mistake once in order to learn from it” in contrast to an ADI (91) item which describes an adaptive response: “As soon as I saw things were not working, I did something about it.” Similarly, an EFI item on impulsivity states, “I take risks, sometimes for fun” in comparison to the state-like description from the ADI: “I couldn't seem to stop moving.” Brief COPE (102) similarly focuses on abstract processes (“I’ve been learning to live with it”), as does the CD-RISC-10 (104) (“I think of myself as a strong person when dealing with life's challenges and difficulties”). These abstractions or mentalizations [e.g., (123)] may be too distal from the intervention activities featured in the JoyPop™ app. Similarly, colleagues who also piloted the JoyPop™ app testing a conditional relationship of change in affect regulation with a DAU variable and ACEs found no significant associations with either the EFI or CD-RISC-10 in a sample of first-year university students (14).

Affect regulation is a central process of stress responsivity adaptive change (15, 44, 46, 55). Our analysis was inspired by the Process Model of Affect Regulation (55) as a framework to explore the dynamic relationship between the four hypothesized domains of resilience activities in the JoyPop™ app intervention design (i.e., Sense-Making, Intervening, Relating, and Visioning, Figure 1) and the psychophysiological and behavioral outcomes of our observed variables of affect regulation, stress responsivity, and social support. Gross et al.'s (55) framework integrates evidence-based stress responsivity processes and mechanisms, the effects of repetition over time, and the complexity of the interaction of stages and strategies of regulation. We analyzed both scales and subscales to explore evidence of engagement in stages and strategies of affect regulation underlying significant change-over-time represented in total marginal mean score scales (Tables 6–8).

In addition to significant change-over-time in total scale scores of the ADI, DERS-SF, PANAS Negative Affect, many subscales were significant as well (Tables 6, 7). All three ADI subscales of affective, behavioral, and cognitive regulation demonstrated significant change-over-time, with the most substantive change in decreased affect dysregulation. Four of the six DERS-SF subscale scores changed significantly, including non-acceptance, goals, impulse, and strategies. The largest effects were in nonacceptance and strategies, both relevant to JoyPop™ features of mood rating and journaling. In qualitative interviews, participants identified these two features as especially helpful (64, 124). Interestingly, although the EFI full scale was not significant, the subscales of organization and impulse control increased (Table 8), as did cognitive regulation processes in both the ADI and DERS-SF, showing significant, if small, change-over-time. Future assessment of the app's efficacy might benefit from including a multi-process measure such as Greenberg et al.'s (123) Mentalized Activity Scale (MAS), which assesses three components of identifying, processing, and expressing emotions to gain nuance in evaluating the JoyPop™ intervention, commensurate with Gross et al.'s (55) model and complex resilience modeling (15, 16, 52, 68).

In the final analysis exploring the mechanisms and processes of change of the app intervention, we examined differential frequency and chronicity of baseline traumatogenic exposure (ACEs), rates of change-over-time, and interaction effects in relation to all eight DVs. The initial GEEs with the five ACE variables did not yield many significant results. However, in the results for one of the two stress responsivity measures (PRSR), significant differences emerged (Table 9). Similar to the ADI and DERS-SF measures, the PRSR is also state-focused with items such as “When I argue with other people…” coupled with option responses “I usually calm down quickly/I usually stay upset for some time/It usually takes me a long time until I calm down” (101). The variables of the aggregate ACEs, chronicity of exposure, and 5+ events were associated with moderate decreases in perceived stress from baseline to post-study, with some significant reductions associated with the indicator TIME and its interaction with ACE variables. These results indicate that consistent use of the JoyPop™ app can reduce stress reactivity (Table 9), especially in groups with high baseline traumatic exposure (Table 2), such as our sample. Our results provide evidence of change-over-time through consistent use of the JoyPop™ app intervention in mechanisms and processes of affect regulation and stress responsivity and social support (minimally, Supplementary Table S2) constructs of the concept of resilience. With 60% of the sample aged 25 years old or younger, these emergent adults are within the crucial window of maturation of the regulation mechanisms of affect and stress reactivity (56–59), which further confirms the relevance of our pilot findings on change-over-time in resilience resulting from consistent JoyPop™ app use as a resilience-enhancing support for adversity-exposed youth.

### 4.1. Limitations

Our pilot study had several important limitations, in addition to those already mentioned. The convenience sample was quite homogenous across gender, sexual identity, ethnicity, and age, and did not include a control group. Although youth extends to approximately age 30 in brain development research [e.g., (117)], 30% of our sample were of adult age. A more complex understanding of the effects of intervention would result from testing with a more diverse and younger sample with more realistic use patterns (i.e., more variation in consistency). Our small sample size and lack of precise data on specific JoyPop™ app feature use frequency and duration inhibited a fine-grained analysis of the relationships between intervention targets and change in underlying mechanisms and processes of resilience. Furthermore, although there is substantial evidence of repetition as the mechanism of change in stress responsivity and affect regulation [e.g., (15, 16, 33, 40, 41, 50, 51)], the amount of time needed to achieve lasting adaptive change through repetition (neuronal rewiring) is not known and likely varies across individuals. A substantial proportion of the change-over-time with app use occurred in the first assessment period, from baseline to mid-study. The evaluation results would be strengthened by longitudinal assessment of the durability of change following the study observation period. A longer observation period may also be necessary to achieve greater magnitude in change, particularly in affect regulation and stress responsivity, patterns of which can become entrenched in early childhood. It is also a challenge to translate small changes in magnitude to real-life effects in resilient functioning and overall well-being. Finally, research on intervention responsivity focused on biological sensitivity to context or environment has demonstrated phenotypic variation in sensitivity to internal and external stressors, which influences sensitivity to interventions (43, 125, 126). Although we explored one indicator of this with the ACEs variables, we were limited with our small sample and lack of DAU variation data to explore intervention responsivity more complexly, the analysis of which would be a strength in future research.

### 4.2. Conclusions and future directions

The JoyPop™ app is a digital intervention designed to enhance resilience for vulnerable youth with exposure to past and current high levels of adversity. The multiple features included in the app were chosen guided by up-to-date evidence on resilience as a complex, dynamic, and multi-process phenomenon amenable to change through repeated engagement in activities (12–14) that target, in particular, stress responsivity, affect regulation, and social connection. Our findings contribute to the ongoing research to bridge the gap between the identification of psychophysiological mechanisms affected by exposure to traumatogenic experiences and the subsequent lived experience of engaging in activities to enhance resilience. With the plethora of digital interventions for mental health, well-being, and resilience avaible, one means to bridge this gap, demonstrated in our study, is the collection of process-focused data. A more fine-grained analysis of specific mechanisms and processes of resilience such as affect regulation could be achieved with multidimensional measures [e.g., MAS, (123)], which test a range of components [e.g., the stages and strategies in the Process Model of Affect Regulation, (55)] and capacity for flexibility in adaptive strategies (17, 65, 67, 127).

Our results suggest that this approach can increase knowledge of which resilience mechanisms and processes the JoyPop™ app is affecting and identify gaps in a complex conceptualization of resilience that the app features may not be influencing directly. For example, in both student sample pilots, findings demonstrated change-over-time at the individual (14) and the group level (this study) in affect regulation, suggesting that the multiple features targeting various aspects of regulation (i.e., rate my mood, diaphragmatic breathing, or journaling) are succeeding as intended. In qualitative interviews, participants confirmed the value of these features for responding to in-the-moment stress and for increasing regulation capacity over time (64, 124). At the same time, our analysis of underlying processes suggests that cognitive processes may be less impacted through app use as demonstrated through a lack of significance on specific measures [i.e., EFI, (14)] or with minimal magnitude on cognition-focused subscales (Tables 6–8). Further exploration is needed to understand if the lack of cognitive change is due to the existing matrix of features within the JoyPop™ app, a function of a mismatch in measurement that is not capturing the cognitive processes being affected by app use, or if our observation period is too short to affect entrenched cognitions resulting from traumatogenic exposure (20–23), taking into account the high levels of ACEs reported by our participants. Future research can assist in clarifying the relationship between app features, use patterns, and change-over-time. Also of interest is the timeframe of change and durability of changes observed in the pilot studies. Post-intervention testing is needed to explore how long app use effects last, particularly given the noticeable pattern of greater magnitude of change seen in the first 2 weeks of the study as compared to the second (see Tables 6–9).

The JoyPop™ app is an evergreen app in that it is responsive to emerging research and will be updated regularly. Indeed, several challenges identified in the pilot research have already been addressed. For example, we have included background information on how included activities can foster resilience, which may encourage a more intentional engagement with app features and increase the app's effectiveness. More psychoeducation for users on features and their intended effects following repeated use can also support use of the app as adjunctive to other services for youth or when access to services, such as mental health support, is costly or limited (128).

Regular use of the JoyPop™ app to change stress response patterns and entrench resilient stress responsivity patterns, i.e., flexible means to reduce or inhibit high arousal states and impulsive behavior, is only achieved through an app that is culturally safe and contextually relevant for a range of users (52, 69, 70). Members of the app development team, informed by research (128) and best practices principles (129), have been collaborating with Indigenous community partners to develop a version of the app that is adapted to be culturally relevant to Haudenosaunnee youth (69, 70, 128, 130).

Although our sample was very homogeneous, the quantitative findings presented here along with our qualitative findings (64) suggest that the app could support resilience for social worker trainees as they enter the profession to manage exposure to high stress work conditions and adjunctively with service users. Working in any helping profession requires both flexibility to adapt to stressors in the moment and over-time adaptive stress responsivity that sustains well-being and resilience (81, 126). Exposure to workplace traumatogenic stressors increases risk of experiencing burnout, compassion fatigue and PTSD (74–77). Given that the high level of prior traumatogenic stressors in our sample is not uncommon (78–81), social work and other helping professions trainees would benefit from access to the JoyPop™ app features to strategically enhance their own resilience. Furthermore, there is benefit in workers experiencing the app themselves, to better understand how they can incorporate it into their practice with service users (64). The app is currently being tested, again with university students, including a randomized control group (131), which will help advance the development of the JoyPop™ app digital intervention and in its preparation for use in communities of practice and by individuals to enhance resilience for vulnerable youth.

## Data Availability

The datasets presented in this article are not readily available because The dataset is still under analysis. Requests to access the datasets should be directed to katherine.maurer@mcgill.ca.
