# Supplementary material for: Building resilience through daily smartphone app use: results of a pilot study of the JoyPop app with social work students
Source: Front Digit Health. 2023 Nov 20;5:1265120. doi: 10.3389/fdgth.2023.1265120 (PMC10694474; doi:10.3389/fdgth.2023.1265120)
Supplement: Supplementary file 1 [file Datasheet1.pdf]

Supplementary Table 1 GEE Estimated marginal means and coefficients for Time and stress responsivity (PTSD, PCL-5) and social support measures (MSPSS) baseline, mid-study, and post-study[a] (n=91)

Time	Mean	β	SE[b]	95% Wald CI[c]	Wald χ^2	*df*[d]	*p* value[e]	Model Effects Wald χ^2	Model Effects *df*[d]	Model Effects *p* value[e]
**PTSD Checklist for DSM-5 (PCL-5)**										
Baseline	21.35		1.65	18.11±24.59						
Mid-study	18.89		1.66	15.63±22.14						
Post-study	17.11		1.63	13.92±20.30						
Intercept		21.35	1.65	18.11±24.59	166.97	1	<.001	172.09	1	<.001
Baseline to mid-study		-2.46	1.30	-5.01±-1.16	3.91	1	.052	7.80[f]	2	.023
Baseline to post-study		-4.24	1.57	-7.31±0.09	7.77	1	.006			
**PCL-5 intrusion symptom subscale**										
Baseline	4.66		0.48	3.72±5.61						
Mid-study	4.21		0.49	3.25±5.18						
Post-study	3.36		0.43	2.52±4.21						
Intercept		4.66	0.48	3.72±5.61	94.30	1	<.001	102.97	1	<.001
Baseline to mid-study		-0.45	0.45	-1.32±0.42	1.20	1	.288	9.89[f]	2	.007
Baseline to post-study		-1.30	0.46	-2.20±-0.39	8.14	1	.004			
**PCL-5 persistent avoidance subscale**										
Baseline	2.49		0.25	2.00±2.98						
Mid-study	1.94		0.23	1.49±2.38						
Post-study	1.62		0.22	1.18±2.05						
Intercept		2.49	0.25	2.00±2.98	98.90	1	<.001	115.08	1	<.001
Baseline to mid-study		-0.55	0.23	-1.00±-0.10	5.79	1	.017	10.07[f]	2	.007
Baseline to post-study		-0.87	0.28	-1.42±-0.33	9.95	1	.002			
**PCL-5 negative alterations subscale**										
Baseline	7.31		0.61	6.11±8.50						
Mid-study	6.53		0.62	5.31±7.75						
Post-study	6.03		0.64	4.78±7.28						
Intercept		7.31	0.61	6.11±8.50	144.01	1	<.001	148.60	1	<.001
Baseline to mid-study		-0.78	0.50	-1.77±-0.21	2.48	1	.119	4.50[f]	2	.108
Baseline to post-study		-1.28	0.62	-2.49±-0.06	4.42	1	.037			
**PCL-5 increased arousal subscale**										
Baseline	5.87		0.43	5.02±6.72						
Mid-study	5.18		0.42	4.36±6.01						
Post-study	5.26		0.47	4.34±6.18						
Intercept		5.87	0.44	5.02±6.73	183.14	1	<.001	218.66	1	<.001
Baseline to mid-study		-0.69	0.39	-1.45±0.08	3.44	1	.072	3.68[f]	2	.170
Baseline to post-study		-0.61	0.46	-1.52±0.30	1.86	1	.179			
**PCL-5 depersonalization subscale**										
Baseline	1.02		0.18	0.66±1.38						
Mid-study	1.03		0.21	0.62±1.43						
Post-study	0.84		0.18	0.48±1.20						
Intercept		1.02	0.18	0.66±1.39	31.06	1	<.001	35.02	1	<.001
Baseline to mid-study		0.00	0.16	-0.31±0.32	0.02	1	.902	1.49[f]	2	.486
Baseline to post-study		-0.19	0.18	-0.53±0.16	1.25	1	.280			

Time	Mean	β	SE[b]	95% Wald CI[c]	Wald χ^2	*df* [d]	*p* value[e]	Model Effects Wald χ^2	Model Effects *df* [d]	Model Effects *p* value[e]
**Multidimensional Scale of Perceived Social Support (MSPSS)**										
Baseline	5.52		0.12	5.29±5.75						
Mid-study	5.56		0.12	5.33±5.79						
Post-study	5.72		0.11	5.50±5.94						
Intercept		5.52	0.12	5.29±5.75	2263.44	1	<.001	3477.74	1	<.001
Baseline to mid-study		0.04	0.12	-0.20±0.28	0.10	1	.751	4.12[f]	2	.128
Baseline to post-study		0.20	0.11	-0.02±0.41	3.32	1	.069			
**MSPSS significant other subscale**										
Baseline	5.66		0.16	5.34±5.97						
Mid-study	5.82		0.14	5.54±6.10						
Post-study	5.96		0.16	5.64±6.27						
Intercept		5.66	0.16	5.34±5.98	1180.41	1	<.001	1901.65	1	<.001
Baseline to mid-study		0.16	0.14	-0.12±0.44	1.24	1	.267	4.36[f]	2	.113
Baseline to post-study		0.30	0.14	0.02±0.58	4.33	1	.038			
**MSPSS family subscale**										
Baseline	5.30		0.16	4.97±5.62						
Mid-study	5.22		0.17	4.89±5.54						
Post-study	5.52		0.16	5.21±5.82						
Intercept		5.30	0.17	4.97±5.63	999.83	1	<.001	1285.90	1	<.001
Baseline to mid-study		-0.08	0.14	-0.36±0.20	0.32	1	.575	7.77[f]	2	.021
Baseline to post-study		0.22	0.12	-0.01±0.45	3.58	1	.059			
**MSPSS friends subscale**										
Baseline	5.61		0.14	5.34±5.88						
Mid-study	5.65		0.13	5.40±5.89						
Post-study	5.69		0.13	5.42±5.95						
Intercept		5.61	0.14	5.34±5.88	1666.21	1	<.001	2596.69	1	<.001
Baseline to mid-study		0.04	0.14	-0.23±0.31	0.08	1	.779	0.35[f]	2	.839
Baseline to post-study		0.08	0.13	-0.18±0.33	0.34	1	.563			

[a] Pooled results of the imputed dataset; [b] Standard error; [c] Confidence interval of the difference; [d] Degrees of freedom; [e] Significant at the .05 level; [f] Test of model effects values for Time.
