# Supplementary material for: Building resilience through daily smartphone app use: results of a pilot study of the JoyPop app with social work students
Source: Front Digit Health. 2023 Nov 20;5:1265120. doi: 10.3389/fdgth.2023.1265120 (PMC10694474; doi:10.3389/fdgth.2023.1265120)
Supplement: Supplementary file 2 [file Datasheet2.pdf]

Supplementary Table 2 GEE Estimated marginal means and coefficients for PSRS social evaluation subscale, Time, and Time x ACEs baseline, mid-study, and post-study[a] (*n*=91)

							Model Effects		
	β	SE[b]	95% Wald CI[c]	Wald X^2	*df*[d]	*p* value[e]	Wald X^2	*df*[d]	*p* value[e]
**PSRS social evaluation subscale**									
Intercept	6.138	0.34	5.47±6.81	319.95	1	<.001	378.01	1	<.001
ACEQ	-0.253	0.09	-0.42±-0.09	8.71	1	.003	14.11[f]	1	<.001
Mid-study	-0.192	0.25	-0.68±0.29	0.60	1	.437	1.87[g]	2	.395
Post-study	-0.442	0.32	-1.07±0.19	1.90	1	.170	0.56[h]	2	.759
Mid-study x ACEQ	-0.044	0.07	-0.18±0.09	0.44	1	.514			
Post-study x ACEQ	-0.051	0.09	-0.22±0.12	0.41	1	.551			
Intercept	6.010	0.32	5.39±6.63	362.88	1	<.001	422.71	1	<.001
ACEchron	-0.156	0.05	-0.26±-0.06	9.18	1	.002	14.03[f]	1	<.001
Mid-study	-0.302	0.25	-0.78±0.18	1.50	1	.218	2.31[g]	2	.315
Post-study	-0.448	0.31	-1.05±0.15	2.17	1	.142	0.81[h]	2	.675
Mid-study x ACEchron	-0.006	0.05	-0.10±0.09	0.02	1	.895			
Post-study x ACEchron	-0.036	0.05	-0.14±0.07	0.56	1	.510			
Intercept	5.299	0.24	4.83±5.77	482.25	1	<.001	545.93	1	<.001
ACEzero	0.246	0.63	-0.99±1.48	0.16	1	.696	0.67[f]	1	.415
Mid-study	-0.368	0.18	-0.71±-0.03	4.55	1	.036	10.24[g]	2	.006
Post-study	-0.682	0.22	-1.11±-0.26	10.05	1	.002	0.95[h]	2	.623
Mid-study x ACEzero	0.223	0.40	-0.56±1.01	0.43	1	.577			
Post-study x ACEzero	0.477	0.58	-0.66±1.62	0.70	1	.411			
Intercept	4.726	0.36	4.01±5.44	170.22	1	<.001	170.81	1	<.001
ACE1-4	1.150	0.44	0.29±2.01	6.81	1	.009	8.74[f]	1	.003
Mid-study	-0.312	0.23	-0.76±0.14	1.82	1	.176	4.80[g]	2	.096
Post-study	-0.632	0.30	-1.22±-0.04	4.70	1	.036	0.24[h]	2	.896
Mid-study x ACE1-4	-0.034	0.32	-0.66±0.59	0.05	1	.914			
Post-study x ACE1-4	0.056	0.41	-0.75±0.86	0.06	1	.892			
Intercept	5.797	0.24	5.34±6.26	608.06	1	<.001	744.48	1	<.001
ACE5+	-1.538	0.50	-2.52±-0.56	9.50	1	.002	15.56[f]	1	<.001
Mid-study	-0.297	0.18	-0.66±0.06	2.63	1	.105	4.25[g]	2	.120
Post-study	-0.487	0.24	-0.97±-0.01	4.07	1	.045	1.00[h]	2	.611
Mid-study x ACE5+	-0.111	0.35	-0.80±0.57	0.10	1	.752			
Post-study x ACE5+	-0.387	0.43	-1.24±0.46	0.88	1	.372			

[a] Pooled results of the imputed dataset; [b] Standard error; [c] Confidence interval of the difference; [d] Degrees of freedom; [e] We used an alpha level of .05; [f] Test of model effects values for ACEs, [g] Test of model effects values for Time; [h] Test of model effects values for Time X ACEs;[I] Dichotomous 11-item self-report measure summed; [j] Chronicity of exposure 11-item self-report measure summed; [k] Indicator variable of zero ACEs reported on ACEQ; [l] Indicator variable of 1-4 ACEs reported on ACEQ; [m] Indicator variable of five or more ACEs reported on ACEQ.
