# Supplementary figures and images for: Building resilience through daily smartphone app use: results of a pilot study of the JoyPop app with social work students
Source: Front Digit Health. 2023 Nov 20;5:1265120. doi: 10.3389/fdgth.2023.1265120 (PMC10694474; doi:10.3389/fdgth.2023.1265120)

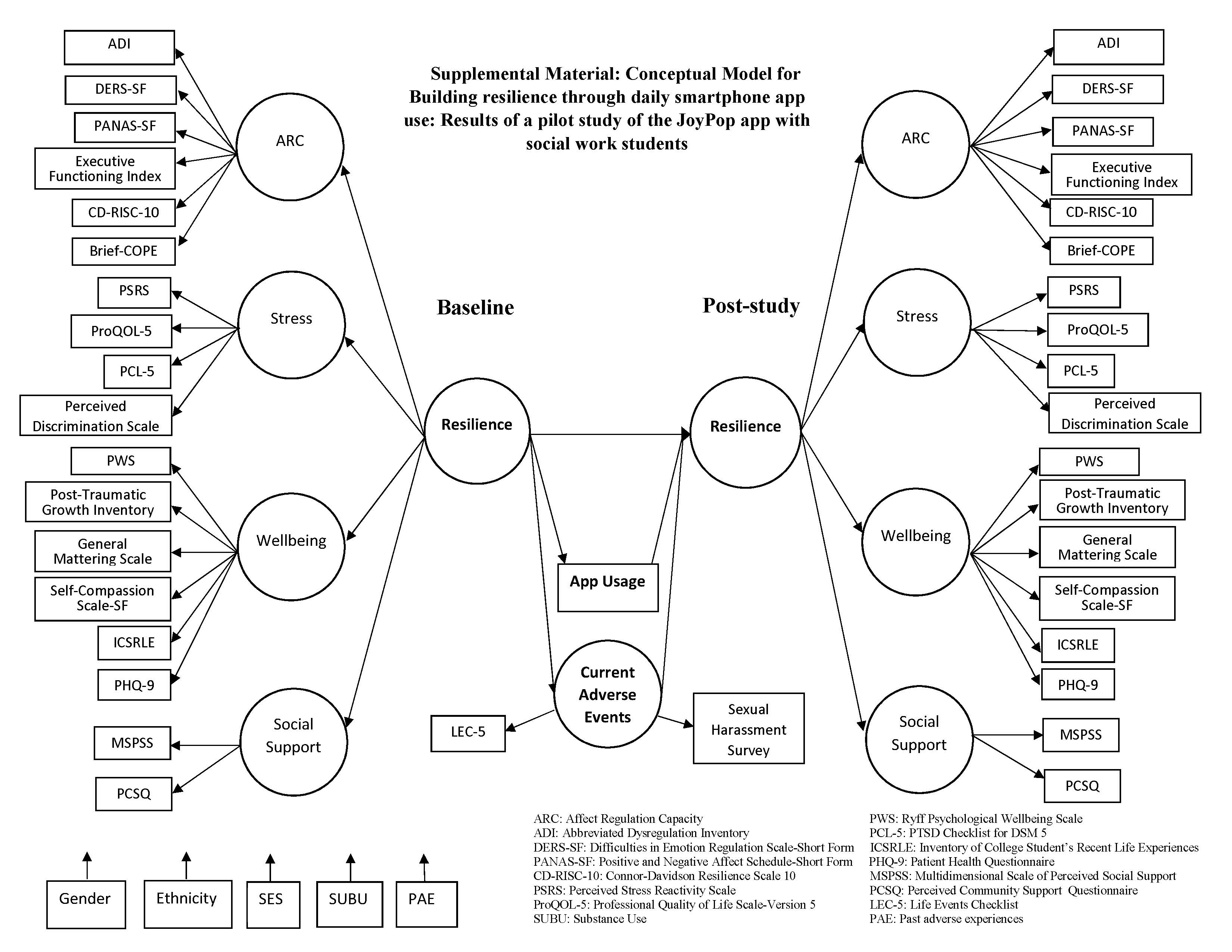

Supplement: Supplementary file 3 [file Image1.jpeg]
